# Exploring *Cassia mimosoïdes* as a promising natural source of steroids with potent anti-cancer, urease inhibition, and antimicrobial properties†

**DOI:** 10.1039/d3ra08913d

**Published:** 2024-03-18

**Authors:** Robert Viani Kepdieu Tchebou, Umar Farooq, Rémy Bertrand Teponno, Tanveer A. Wani, Léon Azefack Tapondjou, Azhar Rasool, Rizwana Sarwar, Aneela Khushal, Syed Majid Bukhari, Seema Zargar, Hong-Guang Xu, Sara Khan

**Affiliations:** a Research Unit of Environmental and Applied Chemistry, Department of Chemistry, Faculty of Science, University of Dschang Box 67 Dschang Cameroon remyteponno@gmail.com; b Department of Chemistry, COMSATS University Islamabad Abbottabad 22010 KPK Pakistan umarf@cuiatd.edu.pk sarakhancuiatd.edu.pk; c Beijing National Laboratory for Molecular Sciences, State Key Laboratory of Molecular Reaction Dynamics, Institute of Chemistry, Chinese Academy of Sciences Beijing 100190 China; d Department of Zoology, GC University Faisalabad Pakistan; e Department of Pharmaceutical Chemistry, College of Pharmacy, King Saud University POBox 2457 Riyadh 11451 Saudi Arabia; f Department of Biochemistry College of Science, King Saud University PO Box 22452 Riyadh 11451 Saudi Arabia

## Abstract

The genus *Cassia* is a rich source of physiologically active secondary metabolites, including a novel compound named 21-methylene-24-ethylidene lophenol, alongside 15 known compounds. These compounds were characterized using different spectroscopic techniques. They exhibited promising antimicrobial activity, particularly against bacteria causing gastrointestinal infections. Compound 1 showed strong anti-bacterial activity against *H. pylori* and *S. aur* with MIC values of 0.28 and 0.12 μg mL^−1^ respectively. The study investigated their impact on *H. pylori*, a contributor to ulcer development, by inhibiting the urease enzyme. Inhibiting urease can reduce *H. pylori*'s pathogenic potential, evident from the fact that the compounds evaluated toward urease enzyme showed higher inhibitory activity (1.024 ± 0.43 <IC_50_ > 6.678±0.11 μM) compared to standard thiourea (IC_50_ = 18.61 ± 0.11 μM). Molecular docking studies confirmed their inhibitory action, with compound 7 notably outperforming thiourea in inhibiting urease (−6.95 kcal mol^−1^*vs.* −3.13 kcal mol^−1^). Additionally, these compounds showed positive effects on liver functioning, which *H. pylori* can impair. Compound 9 shows the best response against human HepG2 liver cancer cell lines *i.e.*, % viability is 14.47% ± 0.69 and IC_50_ is 7.8 μM ± 0.21. These compounds hold potential as lead compounds for addressing gastrointestinal and liver disorders caused by *H. pylori*.

## Abstract 2

The current research discusses the spectroscopic elucidation of the structure and enzymatic activity *in silico* and *in vitro* of fifteen known compounds, as well as a new unidentified avenasterol derivative called 21-methylene-24-ethylidene lophenol, from the genus *Cassia*.

## Graphical abstract

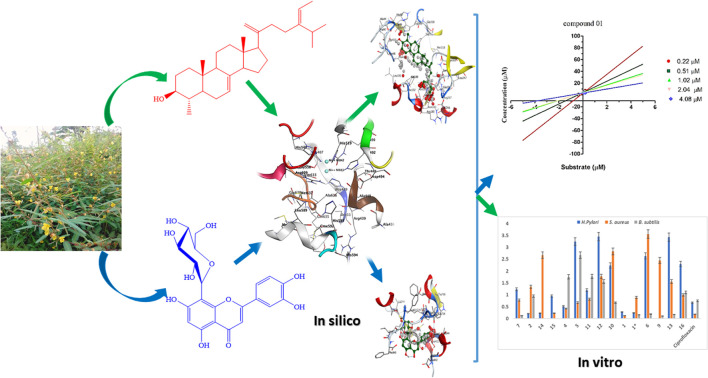

## 1. Introduction

Various medicinal plants have been used for years in daily life to treat diseases around the world. The interest in medicinal plants reflects the recognition of the validity of many traditional claims regarding the value of natural products in health care. *Cassia mimosoides* L. (Caesalpiniaceae), is distributed in various countries including Cameroon, Luzon, Mindanao, India, China, Malaysia, and Australia. This plant is widely used by tribal people to treat various ailments including typhoid fever and other microbial infections.^1,2^ It is used in Uganda to treat pediatric cough.^3^ In northwestern Tanzania, the aerial parts of *C. mimosoides* are pounded and mixed with animal fat, applied topically or taken orally for fractures, cleaning of the uterus by pregnant women, and as an antibacterial. In addition, some South African diviners used these plants for their neurogenic properties.^4^ The roots are used to treat diarrhea, colic, dysentery, and stomach spasms.

Parts of *C. mimosoides* are known to be an important source of secondary metabolites, including anthraquinones. Physcion, chrysophanol, 1,8-dihydroxy-6-methoxy-2-methyl anthraquinone, 1,8-dihydroxy-6-methoxy-3-methyl anthraquinone, and emodin have been reported from the aerial part.^5^ Luteolin, emodin, 1,3-benzenediol, oleanolic acid, (R)-artabotriol, α-l-rhamnose, β-sitosterol, and daucosterol were isolated from an ethanolic extract of *C. mimosoides*.^6^ In addition to phenolic compounds and their derivatives, phytochemical screening of the latter revealed the presence of alkaloids, steroids, saponins, carbohydrates, tannins, glycosides, proteins, and amino acids.^7^

Ureases are common metalloenzymes made by bacteria, fungi, and plants but not by animals. They quickly catalyze the hydrolysis of urea to produce ammonia and carbamate, followed by the urea's breakdown into a second ammonia molecule and carbon dioxide. By giving bacteria nitrogen in the form of ammonia for their growth, the enzyme plays a critical part in their pathogenicity. The pathogenicity of gastrointestinal disorders such as gastritis, duodenal, peptic ulcer, and gastric cancer is significantly influenced by *Helicobacter pylori*'s ureolytic activity. Ammonia produced by ureases is responsible for human and animal cases of hepatic encephalopathy, hepatic coma, urolithiasis, pyelonephritis, and urinary catheter encrustation. In light of this, urease inhibitors have garnered a lot of interest as potential treatments for infections brought on and facilitated by ureolytic activity. Several urease inhibitors, such as fluorofamide, hydroxyureas, and hydroxamic acids have been reported in the past. Some of these inhibitors have, however, been banned from usage *in vivo* because of their instability or toxicity. Active metabolites found in plants are well known to help treat a variety of viral disorders. To address concerns about toxicity right away, a lot of emphasis has been paid to examining the unique biological features of phytochemicals extracted from food plants. It has been demonstrated that many medicinal plants, herbs, extracts, and isolated substances have anti-urease properties.

*Staphylococcus aureus* is an important nosocomial and community-acquired facultative intracellular pathogen. *Staphylococcus aureus*, notorious for causing bacterial infections in liver transplant recipients, often leads to intestinal symptoms.^8^ Meanwhile, *Helicobacter pylori*, a culprit behind peptic ulcers and gastritis, is suspected of playing a role in hepatitis, gallstones, and even hepatobiliary tumors. *Helicobacter pylori*'s ureolytic activity is a game-changer. It significantly impacts gastrointestinal disorders like gastritis, peptic ulcers, and gastric cancer, due to the ammonia it produces. This ammonia also contributes to health problems such as hepatic encephalopathy, hepatic coma, and more.^9,10^

There is therefore a need to identify alternative, safe and less expensive inhibitors for the treatment of these various conditions that undermine the health of populations. In this intricate web of health challenges, the search for novel, affordable, and less toxic treatments is paramount. Medicinal plants like *Cassia mimosoides*, with their arsenal of compounds, hold the potential to revolutionize healthcare, addressing conditions that continue to undermine the well-being of populations. These isolated compounds from nature may just be the key to unlocking safer and more effective treatments for hepatic diseases and beyond.

## 2. Results and discussion

Fifteen compounds were extracted from the ethyl acetate and chloroform fractions of *C. mimosoides*. These compounds underwent purification through column chromatography and HPLC techniques. The resulting pure compounds were then analyzed using ^1^H and ^13^C NMR to determine their chemical structures. Characterization of isolated compounds. Compound 1, obtained as a white powder, exhibited in the SAS-RS-APCI *m*/*Z* 425.42 [M + H]^+^ (positive-ion mode) a molecular formula of C_30_H_49_O.

**Fig. 1:**
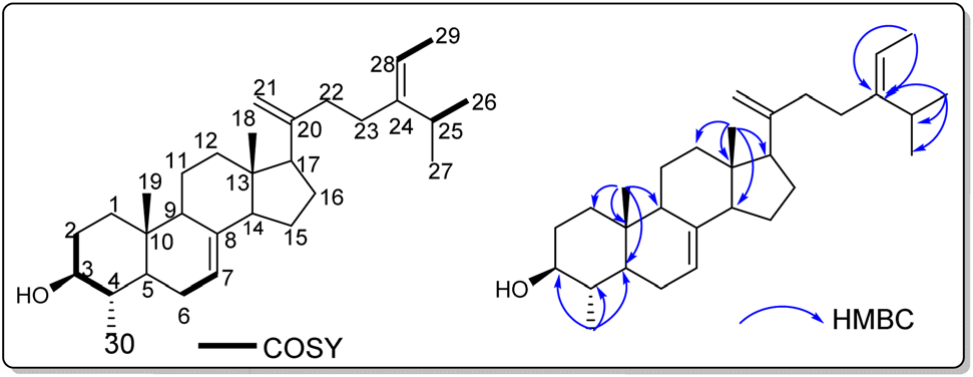
^1^H–^1^H COSY and HMBC correlations of compound 1.

All proton and carbon signals of 1 were assigned on the basis of ^1^H NMR, ^1^H–^1^H COSY, HSQC and HMBC experiments (Table 1). The ^1^H NMR spectrum of compound 1 (Table) exhibited in the up field region signals of two tertiary methyl resonating at *δ*_H_ 0.53 (Me-18) and 0.81 (Me-19), and four secondary methyl at _*δ*_H 1.59 (3H; d; *J* = 6.8 Hz; Me-29), 0.98 (3H; d; *J* = 6.9 Hz; Me-27), 0.97 (3H; d; *J* = 6.5 Hz; Me-26) and at _*δ*_H 0.99 (3H; d; *J* = 6.2 Hz; Me-30). Signals of two olefinic protons were also observed at _*δ*_H 5.17 (1H; dd; *J* = 5.9; 2.3 Hz; H-7) and 5.09 (1H; d; *J* = 6.9 Hz; H-28). In addition, the signals of two methylene protons were observed in this spectrum at _*δ*_H 4.72 (1H; s; H-21a) and 4.66 (1H; t; *J* = 1.7 Hz; H-21b). The ^13^C NMR spectrum of compound 1 combined to the HMBC spectrum showed a set of 30 carbons. The most interesting resonances were those of olefinic carbons depicted at _*δ*_C 117.6 (C-6), 139.1 (C-8), 116.4 (C-28), 148.5 (C-24) and those of the methylene carbons at _*δ*_C 105.8 (C-21), 156.8 (C-20). The backbone of compound 2 was identified as citrostadienol and all the ^1^H and ^13^C NMR spectral data were in good agreement with literature values (Rondet *et al.*, 1999; Schaller, 2010; X. Zhang *et al.*, 2006).^11–13^ Resonances of two methylene protons at _*δ*_H 4.72 (1H; s; H-21a) and 4.66 (1H; t; *J* = 1.7 Hz; H-21b) giving HSQC correlations with the carbon at _*δ*_C 105.8 (C-21) were also observed. The HMBC correlations observed from the protons at _*δ*_H 0.97 (Me-26) and 1.59 (Me-29) to the carbons at _*δ*_C 116.4 (C-28), and 148.5 (C-24) as well as the ^1^H–^1^H COSY correlation between the protons at _*δ*_H 5.17 (H-7) and 2.12 (H-6) allowed us to locate the positions of the double bonds 8 (Fig. 1). Thus, the structure of 1 was established as 21-methylene-24-ethylidene lophenol, a previously unreported avenasterol-type phytosterols to which we gave the trivial name 21citrostadienol.

**Table 1:** ^13^C NMR (100 MHz) and ^1^H NMR (400 MHz) data of compound 1 (CDCl_3_): *δ* in ppm, *J* in Hz

Positions	*δ* ^13^C	*δ* ^1^H (mult, *J*)
1	36.9	1.84 (m, 1H)
		1.81 (m, 1H)
2	30.9	1.80 (m, 1H)
		1.46 (m, 1H)
3	76.2	3.12 (m, 1H)
4	40.2	1.30 (m, 1H)
5	46.6	1.01 (m, 1H)
6	26.6	2.13 (m, 1H)
		2.09 (m, 1H)
7	117.6	5.17(dt, *J* = 5.9, 2.3 Hz, 1H)
8	139.1	—
9	49.6	1.64 (m, 1H)
10	34.8	—
11	21.3	1.55 (m, 1H)
		1.45 (m, 1H)
12	39.4	2.02 (m, 1H)
		1.22 (m, 1H)
13	43.3	—
14	54.9	1.80 (m, 1H)
15	22.8	1.51 (m, 1H)
		1.25 (m, 1H)
16	29.6	1.25 (m, 1H)
17	55.9	1.22 (m, 1H)
18	11.8	0.53 (s, 3H)
19	14.1	0.83 (s, 3H)
20	156.8	—
21	105.8	4.72 (s, 1H)
		4.66 (t, *J* = 1.7 Hz)
22	29.6	1.25 (m, 1H)
23	27.9	1.91 (m, 1H)
		1.27 (m, 1H)
24	145.8	—
25	28.5	2.85 (m, 1H)
26	21.0	0.97 (d, *J* = 6.5 Hz, 3H)
27	20.9	0.98 (d, *J* = 6.9 Hz, 3H)
28	116.4	5.09 (q, *J* = 6.9 Hz, 1H)
29	12.7	1.59 (d, *J* = 6.8 Hz, 3H)
30	15.1	0.99 (d, *J* = 6.2 Hz, 3H)

**Fig. 2:**
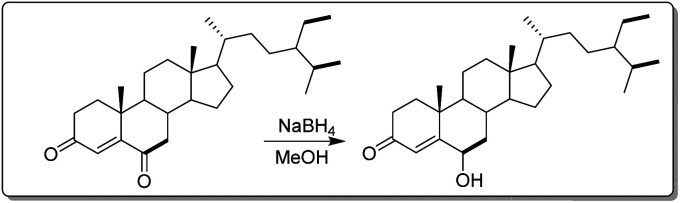
Synthetic scheme semi-derivative of stigmast-4-en-3,6-dione.

### 2.1. Luteolin^1^

Yellow powder, ^1^H NMR (600 MHz, Acetone-d6) *δ* (ppm): 6.59 (1H, s, H-3), 12.99 (1H, s, OH-5), 6.26 (1H, d, *J* = 2.1 Hz, H-6), 6.53 (1H, d, *J* = 2.3 Hz, H-8), 7.47 (1H, dd, *J* = 8.4, 2.2 Hz, H-2′), 7.01 (1H, d, *J* = 8.4 Hz, H-3′), 7.50 (1H, d, *J* = 2.2 Hz, H-6′). ^13^C NMR (150 MHz, Acetone-d6) *δ* (ppm): 164.3 (C-2), 103.1 (C-3), 182.2 (C-4), 162.3 (C-5), 98.8 (C-6), 164.2 (C-7), 93.8 (C-8), 157.8 (C-9), 104.3 (C-10), 122.6 (C-1′), 119.1 (C-2′), 115.6 (C-3′), 149.4 (C-4′), 145.7 (C-5′), 113.0 (C-6′).

### 2.2. 3′,4′,7-Trihydroxyflavone^2^

Yellow powder, ^1^H NMR (600 MHz, Acetone-d6) *δ* (ppm): 6.64 (1H, s, H-3), 7.62 (1H, d, *J* = 8.4 Hz, H-5), 6.79 (1H, dd, *J* = 8.4, 2.0 Hz, H-6), 6.83 (1H, d, *J* = 2.0 Hz, H-8), 7.35 (1H, dd, *J* = 8.3, 2.1 Hz, H-2′), 6.95 (1H, d, *J* = 8.2 Hz, H-3′), 7.58 (1H, d, *J* = 2.1 Hz, H-6′). ^13^C NMR (150 MHz, Acetone-d6) *δ* (ppm): 146.3 (C-2), 111.3 (C-3), 181.5 (C-4), 125.5 (C-5), 114.0 (C-6), 168.0 (C-7), 98.5 (C-8), 165.7 (C-9), 112.5 (C-10), 117.7 (C-1′), 124.7 (C-2′), 115.6 (C-3′), 147.3 (C-4′), 145.2 (C-5′), 124.5 (C-6′).

### 2.3. Luteolin-5-methyl ether^3^

Yellow powder, ^1^H NMR (400 MHz, CD3OD) *δ* (ppm): 6.47 (1H, s, H-3), 6.40 (1H, d, *J* = 2.2 Hz, H-6), 6.53 (1H, d, *J* = 2.1 Hz, H-8), 7.33 (1H, dd, *J* = 8.9, 2.3 Hz, H-2′), 6.89 (1H, d, *J* = 8.9 Hz, H-3′), 7.30 (1H, d, *J* = 2.3 Hz, H-6′), 3.88 (3H, s, OCH3-5). ^13^C NMR (100 MHz, CD3OD) *δ* (ppm): 162.4 (C-2), 105.1 (C-3), 178.8 (C-4), 161.0 (C-5), 96.1 (C-6), 163.6 (C-7), 94.9 (C-8), 159.8 (C-9), 106.8 (C-10), 122.2 (C-1′), 118.5 (C-2′), 115.3 (C-3′), 149.1 (C-4′), 145.6 (C-5′), 112.5 (C-6′), 55.0 (OCH3-5).

### 2.4. Apigenin-8-C-β-d-glucopyranoside^1^

Yellow powder, ^1^H NMR (400 MHz, DMSO-d6) *δ* (ppm): 6.77 (1H, s, H-3), 13.17 (1H, s, OH-5), 6.26 (1H, s, H-6), 8.02 (2H, d, *J* = 8.02, H-2′/H-6′), 6.88 (2H, d, *J* = 8.02 Hz, H-3′/H-5′), 4.67 (1H, dd, *J* = 9.8, 3.4 Hz, H-1′′), 3.82 (1H, dd, *J* = 9.8, 3.4 Hz, H-2′′), 3.25 (1H, m, H-3′′), 3.36 (1H, m, H-4′′), 3.23 (1H, m, H-5′′), 3.76 (1H, d, *J* = 12.1 Hz, H-6a′′), 3.52 (1H, d, *J* = 6.4 Hz, H-6b''). ^13^C NMR (100 MHz, DMSO-d6) *δ* (ppm): 164.6 (C-2), 102.9 (C-3), 182.5 (C-4), 161.0 (C-5), 98.6 (C-6), 163.1 (C-7), 105.2 (C-8), 156.7 (C-9), 104.6 (C-10), 122.6 (C-1′), 129.4 (C-2'/C-6′), 116.2 (C-3′/C-5′), 73.9 (C-1′′), 71.2 (C-2′′), 79.1 (C-3′′), 70.9 (C-4′′), 82.3 (C-5′′), 61.7 (C-6′′).

### 2.5. Luteolin-8-C-β-d-glucopyranoside (orientin)^1^

Yellow powder, ^1^H NMR (400 MHz, DMSO-d6) *δ* (ppm): 6.64 (1H, s, H-3), 13.17 (1H, s, OH-5), 6.25 (1H, s, H-6), 7.52 (1H, dd, *J* = 8.5, 2.2 Hz, H-2′), 6.87 (1H, d, *J* = 8.4 Hz, H-3′), 7.46 (1H, d, *J* = 2.2 Hz, H-6′), 4.67 (1H, dd, *J* = 9.8, 3.4 Hz, H-1′′), 3.83 (1H, m, H-2′′), 3.24 (1H, m, H-3′′), 3.36 (1H, m, H-4′′), 3.28 (1H, m, H-5′′), 3.77 (1H, m, H-6a′′), 3.53 (1H, m, H-6b′′). ^13^C NMR (100 MHz, DMSO-d6) *δ* (ppm): 164.6 (C-2), 102.8 (C-3), 182.4 (C-4), 160.7 (C-5), 98.6 (C-6), 162.9 (C-7), 104.9 (C-8), 156.4 (C-9), 104.4 (C-10), 122.3 (C-1′), 119.8 (C-2′), 116.0 (C-3′), 150.1 (C-4′), 146.2 (C-5′), 114.4 (C-6′), 73.8 (C-1′′), 71.2 (C-2′′), 79.1 (C-3′′), 71.0 (C-4′′), 82.3 (C-5′′), 61.7 (C-6′′).

### 2.6. Luteolin-6-C-β-d-glucopyranoside^14^

Yellow powder, ^1^H NMR (400 MHz, DMSO-d6) *δ* (ppm): 6.70 (1H, s, H-3), 13.58 (1H, s, OH-5), 6.50 (1H, s, H-8), 7.44 (1H, dd, J = 8.2, 2.3 Hz, H-2′), 6.87 (1H, d, *J* = 8.3 Hz, H-3′), 7.42 (1H, d, *J* = 2.3 Hz, H-6′), 4.60 (1H, d, *J* = 9.8 Hz, H-1′′), 4.07 (1H, d, *J* = 9.2 Hz, H-1′′), 3.21 (1H, m, H-3′′), 3.14 (1H, m, H-4′′), 3.18 (1H, m, H-5′′), 3.70 (1H, m, H-6a′′), 3.42 (1H, m, H-6b''). ^13^C NMR (100 MHz, DMSO-d6) *δ* (ppm): 164.0 (C-2), 103.7 (C-3), 182.3 (C-4), 161.2 (C-5), 109.3 (C-6), 163.7 (C-7), 93.9 (C-8), 156.7 (C-9), 103.9 (C-10), 121.9 (C-1′), 116.4 (C-2′), 116.0 (C-3′), 150.1 (C-4′), 146.2 (C-5′), 114.4 (C-6′), 73.4 (C-1′′), 70.6 (C-2′′), 79.4 (C-3′′), 71.0 (C-4′′), 82.0 (C-5′′), 61.7 (C-6′′).

### 2.7. Butin^4^

An orange powder, ^1^H NMR (600 MHz, Acetone-d6) *δ* (ppm): 5.40 (1H, dd, *J* = 12.8, 2.9 Hz, H-2), 3.02 (1H, dd, *J* = 16.7, 12.8 Hz, H-3a), 2.68 (1H, dd, *J* = 16.7, 3.0 Hz, H-3b), 7.73 (1H, d, *J* = 8.6 Hz, H-5), 6.58 (1H, dd, *J* = 8.6, 2.3 Hz, H-6), 6.43 (1H, d, *J* = 2.3 Hz, H-8), 7.05 (1H, d, *J* = 1.9 Hz, H-2′), 6.87 (1H, d, *J* = 8.1 Hz, H-5′), 6.89 (1H, dd, *J* = 8.2, 1.9 Hz, H-6′). ^13^C NMR (150 MHz, Acetone-d6) *δ* (ppm): 79.6 (C-2), 43.8 (C-3), 189.6 (C-4), 128.5 (C-5), 110.2 (C-6), 163.5 (C-7), 102.7 (C-8), 163.2 (C-9), 114.3 (C-10), 131.2 (C-1′), 113.7 (C-2′), 145.6 (C-3′), 145.6 (C-4′), 114.7 (C-5′), 118.2 (C-6′).

### 2.8. Stigmast-4-en-3,6-dione^5^

White powder, ^1^H NMR (600 MHz, C5D5N) *δ* (ppm): 1.69 (1H, m, H-1a), 1.52 (1H, m, H-1b), 1.78 (1H, m, H-2a), 1.39 (1H, m, H-2b), 6.41 (1H, s, H-4), 2.67 (1H, dd, *J* = 16.1, 4.4 Hz, H-7a), 2.05 (1H, dd, *J* = 16.1, 12.3 Hz, H-7b), 1,78 (1H, m, H-8), 1.24 (1H, m, H-9), 1.48 (2H, m, H-11), 2.00 (1H, m, H-12a), 1,15 (1H, m, H-12b), 1.02 (1H, m, H-14), 1.51 (1H, m, H-15a), 1.31 (1H, m, H-15b), 1.83 (2H, m, H-16), 1.03 (1H, m, H-17), 0.68 (3H, s, H-18), 1.00 (3H, s, H-19), 1.38 (1H, m, H-20), 0.98 (3H, d, *J* = 6.3 Hz, H-21), 2.39 (1H, m, H-22a), 1,05 (1H, m, H-22b), 1.24 (2H, m, H-23), 1.49 (1H, m, H-24), 1.68 (1H, m, H-25), 0.88 (3H, d, *J* = 6.1 Hz, H-26), 0.85 (3H, d, *J* = 6.8 Hz, H-27), 1.31 (2H, m, H-28), 0.90 (3H, t, H-29). ^13^C NMR (150 MHz, C5D5N) *δ* (ppm): 35.1 (C-1), 33.9 (C-2), 198.9 (C-3), 125.3 (C-4), 160.8 (C-5), 201.6 (C-6), 46.5 (C-7), 39.1 (C-8), 50.4 (C-9), 34.0 (C-10), 20.7 (C-11), 39.5 (C-12), 42.4 (C-13), 55.8 (C-14), 23.1 (C-15), 28.1 (C-16), 56.2 (C-17), 11.7 (C-18), 16.9 (C-19), 36.0 (C-20), 18.6 (C-21), 33.8 (C-22), 26.1 (C-23), 45.8 (C-24), 29.2 (C-25), 19.3 (C-26), 18.9 (C-27), 23.1 (C-28), 11.9 (C-29).

### 2.9. Stigmast-4-en-3β,6α-diol (Zhao *et al.*,^15^ 2005)

White powder, ^1^H NMR (400 MHz, Acetone-d6) *δ* (ppm): 1.73 (1H, m, H-1a), 1.69 (1H, m, H-1b), 1.40 (1H, m, H-2a), 1.27 (1H, m, H-2b), 4.08 (1H, m, H-3), 5.74 (1H, d, *J* = 1.7 Hz, H-4), 4.11 (1H, m, H-6), 2.02 (2H, m, H-7), 1,30 (1H, m, H-8), 0.72 (1H, m, H-9), 1.50 (1H, m, H-11a), 1.38 (1H, m, H-11b), 2.05 (1H, m, H-12a), 1,16 (1H, m, H-12b), 1.17 (1H, m, H-14), 1.23 (2H, m, H-15), 1.69 (2H, m, H-16), 1.05 (1H, m, H-17), 1.05 (3H, s, H-18), 0.74 (3H, s, H-19), 1.42 (1H, m, H-20), 0.96 (3H, d, *J* = 6.4 Hz, H-21), 1.67 (1H, m, H-22a), 1,40 (1H, m, H-22b), 1.92 (2H, m, H-23), 0.98 (1H, m, H-24), 1.70 (1H, m, H-25), 0.88 (3H, m, H-26), 0.84 (3H, m, H-27), 1.30 (2H, m, H-28), 0.88 (3H, m, H-29). ^13^C NMR (100 MHz, Acetone-d6) *δ* (ppm): 36.4 (C-1), 34.3 (C-2), 67.4 (C-3), 121.5 (C-4), 147.5 (C-5), 67.0 (C-6), 42.5 (C-7), 29.5 (C-8), 54.5 (C-9), 37.4 (C-10), 20.8 (C-11), 39.7 (C-12), 42.4 (C-13), 56.1 (C-14), 25.8 (C-15), 24.0 (C-16), 56.0 (C-17), 19.2 (C-18), 11.4 (C-19), 36.0 (C-20), 18.2 (C-21), 33.7 (C-22), 28.0 (C-23), 45.8 (C-24), 29.1 (C-25), 19.2 (C-26), 18.4 (C-27), 22.8 (C-28), 11.3 (C-29).

### 2.10. Emodin^6^

Orange powder, ^1^H NMR (600 MHz, Acetone-d6) *δ* (ppm): 12.10 (1H, s, OH-1), 7.15 (1H, s, H-2), 7.58 (1H, d, *J* = 1.6 Hz, H-4), 7.26 (1H, d, *J* = 2.4 Hz, H-5), 6.67 (1H, d, *J* = 2.4 Hz, H-7), 12.20 (1H, s, OH-8), 2.48 (3H, s, H-1′). ^13^C NMR (150 MHz, Acetone-d6) *δ* (ppm): 165.4 (C-1), 124.0 (C-2), 148.6 (C-3), 120.5 (C-4), 133.3 (C-4a), 108.9 (C-5), 162.3 (C-6), 107.9 (C-7), 165.9 (C-8), 109.1 (C-8a), 190.7 (C-9), 113.6 (C-9a), 181.4 (C-10), 135.6 (C-10a), 21.0 (C-1′).

### 2.11. Chrysophanol^7^

Orange powder, ^1^H NMR (600 MHz, CDCl3) *δ* (ppm): 12.10 (1H, s, OH-1), 7.12 (1H, m, H-2), 7.67 (1H, d, *J* = 1.8 Hz, H-4), 7.84 (1H, dd, *J* = 7.5, 1.2 Hz, H-5), 7.69 (1H, d, *J* = 8.0 Hz, H-6), 7.31 (1H, dd, *J* = 8.5, 1.2 Hz, H-7), 12.03 (1H, s, OH-8), 2.49 (3H, s, H-1′). ^13^C NMR (150 MHz, CDCl3) *δ* (ppm): 162.4 (C-1), 124.5 (C-2), 149.3 (C-3), 121.3 (C-4), 133.2 (C-4a), 119.9 (C-5), 136.9 (C-6), 124.3 (C-7), 162.7 (C-8), 115.8 (C-8a), 192.5 (C-9), 113.7 (C-9a), 182.0 (C-10), 133.6 (C-10a), 22.2 (C-1′).

### 2.12. Physcion^6^

**Fig. 3:**
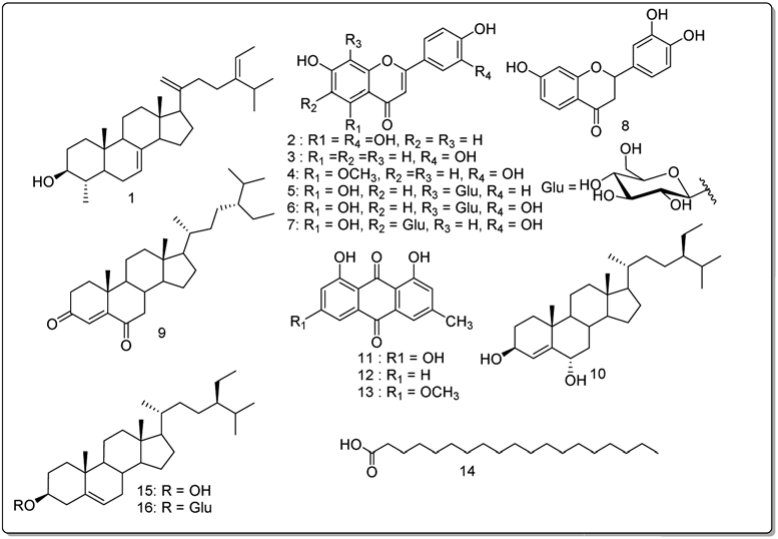
Structures of compounds (1–16) isolated from *Cassia mimosoïdes*.

Orange powder, ^1^H NMR (400 MHz, CDCl3) *δ* (ppm): 12.13 (1H, s, OH-1), 7.09 (1H, d, *J* = 1.5 Hz, H-2), 7.63 (1H, d, *J* = 1.7 Hz, H-4), 7.37 (1H, d, *J* = 2.6 Hz, H-5), 6.69 (1H, d, *J* = 2.5 Hz, H-7), 12.33 (1H, s, OH-8), 2.46 (3H, s, H-1′), 3.94 (3H, s, H-1′′). ^13^C NMR (100 MHz, CDCl3) *δ* (ppm): 162.5 (C-1), 124.5 (C-2), 148.4 (C-3), 121.3 (C-4), 133.2 (C-4a), 108.2 (C-5), 166.5 (C-6), 106.7 (C-7), 165.2 (C-8), 110.2 (C-8a), 190.8 (C-9), 113.7 (C-9a), 181.8 (C-10), 135.2 (C-10a), 22.1 (C-1′), 56.1 (C-1′′) (Fig. 3).

These compounds were tested for their antimicrobial activity, especially against bacteria that cause gastrointestinal infections. The study also evaluates their impact on *H. pylori*, a bacterium associated with ulcers, and identifies potential benefits through the inhibition of urease, a critical enzyme linked to ulcer development. Molecular docking analysis confirms the structure–activity relationship, demonstrating robust urease inhibition through various interactions, surpassing the standard thiourea. Furthermore, the compounds undergo testing on Hep G2 cells, indicating potential positive effects on liver function, given that *H. pylori* can disrupt liver function. These compounds offer targeted solutions for gastrointestinal and liver issues associated with *H. pylori* infection. These findings suggest that further optimization could lead to the development of these compounds as lead candidates for therapeutic purposes. The results follow as under.

### 2.13. *In vitro* analysis

#### 2.13.1. Anti-microbial assays

Anti-microbial assays of the isolated componds were performed against the selected strain of fungi *i.e.*, *Alternaria alternata*, *Aspergillus fumigatus*, *Aspergillus niger*, and *Penicillium citrii*. MIC-value were taken in μM mL^−1^ of the compounds and these compounds show different modes of inhibition against different strains of fungi (Table 2). Emodin (compound 11), chrysophenol (compound 12), physcion (compound 13) and stigmast-4-en-3,6-dione (compound 9) show the best inhibition potential against *Alternaria alternata* strain. Luteolin (compound 2), luteolin-8-C-β-d-glucopyranoside (compound 6), stigmast-4-en-3β,6α-diol (compound 10), stigmast-4-en-3,6-dione (compound 9) and apigenin-8-C-β-d-glucopyranoside (compound 5) show more potential inhibition against *Aspergillus fumigatus* as their MIC-values are lower than other compounds. Luteolin-5-methyl ether (compound 4), apigenin-8-C-β-d-glucopyranoside (compound 5), luteolin-8-C-β-d-glucopyranoside (compound 6) and stigmast-4-en-3β,6α-diol (compound 10) show lower MIC-value as their inhibition much more than rest of the compounds against *Aspergillus niger*. Apigenin-8-C-β-d-glucopyranoside (compound 5), luteolin-8-C-β-d-glucopyranoside (compound 6), 3′,4′,7-trihydroxy flavone (compound 3) and butin (compound 8) are more potent inhibitor of *Penicillium citrii*.

**Table 2:** MIC values (μM) of the isolated compounds against *Alternaria alternata*, *Aspergillus fumigatus*, *Aspergillus niger*, and *Penicillium citrii* strains

Sample	*Alternaria alternata*	*Aspergillus fumigatus*	*Aspergillus niger*	*Penicillium citrii*
Luteolin	0.043 ± 0.002	0.01 ± 0.004	0.021 ± 0.001	0.05 ± 0.002
3′,4′,7-Trihydroxy flavone	0.056 ± 0.001	0.013 ± 0.003	0.032 ± 0.005	0.016 ± 0.01
Luteolin-5-methyl ether	0.03 ± 0.006	0.04 ± 0.002	0.01 ± 0.006	0.06 ± 0.004
Apigenin-8-C-β-d-glucopyranoside	0.025 ± 0.007	0.01 ± 0.006	0.015 ± 0.001	0.01 ± 0.006
Luteolin-8-C-β-d-glucopyranoside	0.021 ± 0.005	0.014 ± 0.005	0.013 ± 0.006	0.015 ± 0.008
Luteolin-6-C-β-d-glucopyranoside	0.06 ± 0.007	0.25 ± 0.003	0.0072 ± 0.0003	0.022 ± 0.003
Butin	0.02 ± 0.006	0.017 ± 0.005	0.005 ± 0.0003	0.10 ± 0.003
Stigmast-4-en-3,6-dione	0.15 ± 0.005	0.125 ± 0.0015	0.02 ± 0.006	0.04 ± 0.003
Stigmast-4-en-3β,6α-diol	0.045 ± 0.003	0.01 ± 0.006	0.015 ± 0.005	0.20 ± 0.03
Emodin	0.01 ± 0.006	0.035 ± 0.005	0.04 ± 0.003	0.045 ± 0.005
Chrysophanol	0.012 ± 0.003	0.06 ± 0.001	0.03 ± 0.002	0.05 ± 0.001
Physcion	0.015 ± 0.005	0.035 ± 0.006	0.09 ± 0.009	0.03 ± 0.005

#### 2.13.2 Antibacterial activity

**Fig. 4:**
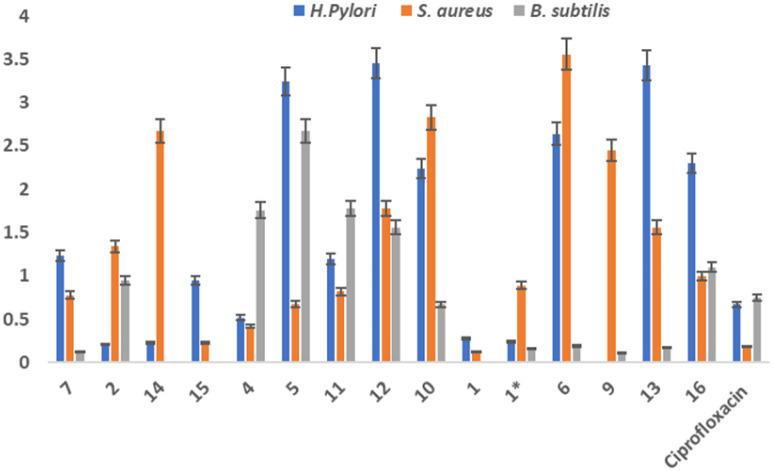
Antibacterial activity of isolated compounds against bacterial strains. *H.pylori*, *B.subtilis* and *S.aureus*. (1* = synthesized compound).

Antibacterial activity of the isolated and synthesized compounds was perform using agar well method. Three bacterial strains selected for this experiment were *H.pylori*, *B. subtilis* and *S. aureus*, for all of three strains compounds show good to mederate inhibition. Some compounds show best inhibition potential as their minimum inhibition concentrations (MIC) are less than strandard drug (ciprofloxacin). The presence of *H. pylori* in the stomach has been associated with a range of gastric disorders, including peptic ulcers, gastritis, and an increased risk of gastric cancer. The bacterium's ability to manipulate the gastric environment through urease activity underscores its role in the development and progression of these conditions. Different studies have targeted *H. pylori* and its urease activity as potential avenues for therapeutic intervention and disease management (Fig. 4).

#### 2.13.3 Urease inhibition potential

The inhibition potential of the isolated compounds against urease was investigated using a SpectraMax M2 reader using 96-well microplates. Results indicated that most examined compounds demonstrated inhibition of the urease enzyme. Inhibition percentages ranged from 53.42 to 83.05% (Table 3). Notably, the *in vitro* urease inhibition assay data highlighted the significance of secondary metabolite type and substitution pattern in influencing the structure–activity relationship against urease. In terms of inhibitory concentrations, the evaluated compounds displayed a range from 1.02 ± 0.35 to 6.678 ± 0.11 μM (Table 3), with compounds 1, 7, 2, and 14 showing the most favorable inhibitory concentrations. Worth noting, all assessed compounds exhibited higher inhibitory concentrations than the reference compound. These findings underscore the potential of the investigated compounds as promising inhibitors of urease, with their inhibitory concentrations providing insights into their effectiveness against the enzyme.

**Table 3:** *In vitro* study % enzyme inhibition and IC_50_ of isolated compounds

Compounds	% Inhibition	IC_50_ ± SEM (μM)
7	81.57	1.22 ± 0.43
2	82.05	1.30 ± 0.28
14	80.42	1.95 ± 0.31
15	80.00	2.78 ± 0.24
4	82.73	3.87 ± 0.24
5	77.52	4.73 ± 0.22
11	72.89	5.20 ± 0.14
12	78.21	5.92 ± 0.25
10	76.57	6.68 ± 0.11
1	82.32	1.02 ± 0.35
Synthesized compound	69	8.03 ± 0.19
6	71.52	5.03 ± 0.12
9	13.42	—
13	63.47	6.24 ± 0.30
16	19.47	—
Standard (thiourea)	86.73	18.61 ± 0.11

**Fig. 5:**
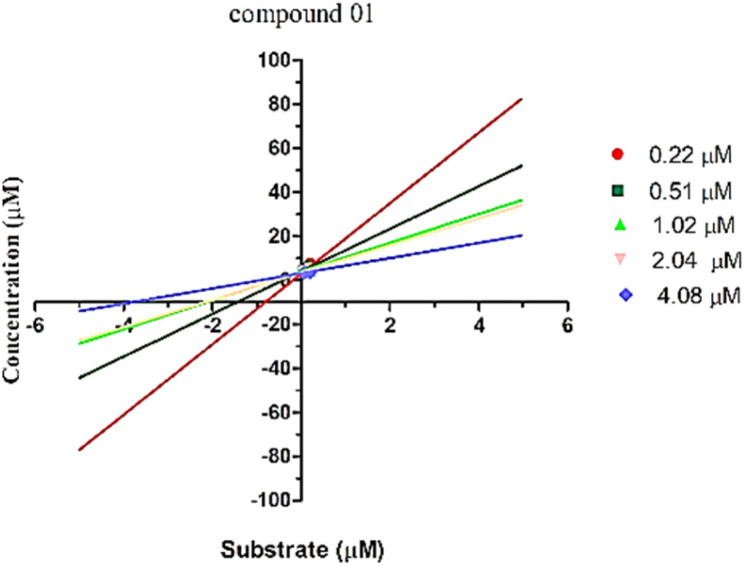
Lineweaver–Burk plot for the compound 1.

The enzyme kinetic analysis focused on the most potent inhibitor identified, compound 1, against urease. The kinetic assay involved preparing varying concentrations of both the test compound and the substrate (urea), spanning 0 μM, 5 μM, 10 μM, 15 μM, and 20 μM. The resulting data was utilized to construct a Lineweaver–Burk plot. The Lineweaver–Burk plot revealed a competitive mode of inhibition for compound 1 (Fig. 5). This observation suggests a scenario where both the substrate and compound 1 compete for the enzyme's active site. This competitive relationship signifies that compound 1 effectively occupies the enzyme's active pocket, which aligns with the outcomes of the molecular docking study that also indicated a robust binding of compound 1 within the enzyme's active site.

**Fig. 6:**
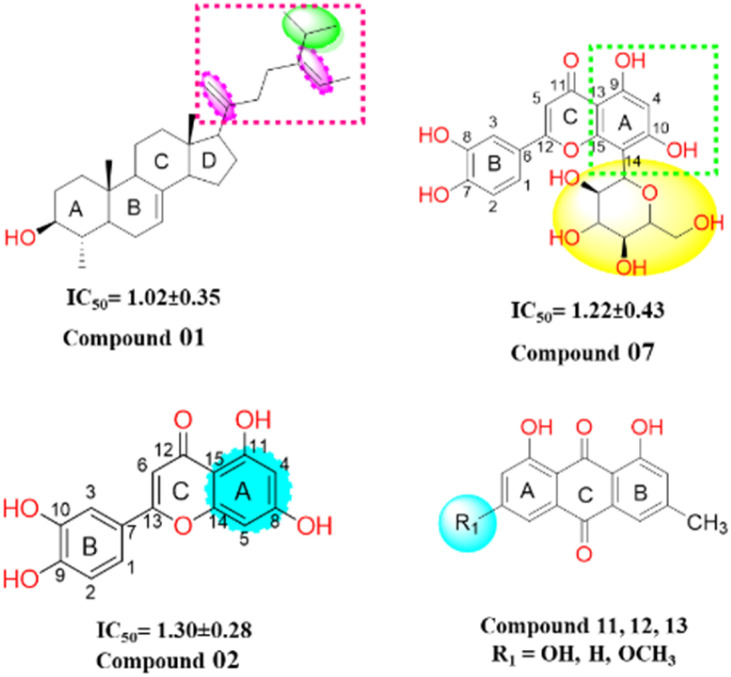
Structure of potent compounds with their IC_50_ -values.

Structure–activity relationship (SAR) analysis proves invaluable in discerning the structural attributes pivotal for the efficacy of the isolated compounds and their inhibitory potential against the target enzyme (Fig. 6). Examination of the inhibitory outcomes (as presented in Table 3) discloses that among the entire array of compounds studied, compounds 1, 2, and 7 exhibit the most potent inhibition, showcasing impressive IC_50_ values of 1.02 μM, 1.30 μM, and 1.22 μM, respectively. These compounds stand out due to the presence of olefinic, alkyl, and hydroxyl groups, which contribute to heightened lipophilicity and hydrophilicity. An in-depth SAR analysis of compound 1 reveals that the substitution of 5-isopropyl-2-methylhepta-1,5-diene at ring D (a five-membered ring) emerges as a crucial determinant of biological activity. This substitution notably enhances lipophilicity, thereby exerting a substantial impact on the observed inhibition. Additionally, hydroxyl and methyl moieties at the juncture of ring A, B, and ring C, D, along with those at ring A, play pivotal roles in the urease inhibition process.

In compound 2, the introduction of hydroxyl groups at positions 8 and 11 in ring A, and positions 9 and 10 in ring B, leads to a notable enhancement in inhibitory activity against the urease enzyme. This effect is attributed to the electron-withdrawing nature of the hydroxyl group from the benzene moieties, which increases the compounds' hydrophilicity by facilitating hydrogen bonding interactions. Compound 7 similarly exhibits effective inhibition, displaying even greater potency compared to compound 2. This increased potency can be attributed to the substitution of the sugar moiety at position (C 14) of the flavonoid structure. Among the remaining compounds (14, 15, 4, 5, 11, 12, 10, 6, and 13), a range of good to moderate inhibition is observed, with corresponding IC_50_ values of 1.95, 2.78, 3.87, 4.73, 5.20, 5.92, 6.68, 5.03, and 6.24 μM, respectively. However, compounds 9 and 16 exhibit weaker inhibition against the urease enzyme, with inhibition percentages of 13.42% and 19.47%, respectively. Notably, in anthraquinones (compounds 11, 12, and 13), the presence of a hydroxyl or a methoxy group on the A ring diminishes their inhibitory potency. In the case of steroids, the inhibitory efficacy is contingent upon the steroid skeleton's nature and its specific substitutions. For instance, the substitution of a glucose unit for a hydrogen in sterol (compounds 15 and 16) significantly reduces its inhibitory potential against the urease enzyme. Furthermore, intriguing insights emerge from the reduction of the ketone function in compound 9 to an alcohol (compound 10), which substantially enhances its inhibitory activity. These findings are reinforced by *in vitro* docking studies, where all isolated compounds displayed strong binding energies in the active site, particularly the potent inhibitors. Moreover, the kinetic studies of the novel isolated compound affirmed a competitive mode of inhibition, adding further depth to our understanding of its inhibitory mechanism.

### 2.14 Molecular docking studies

**Fig. 7:**
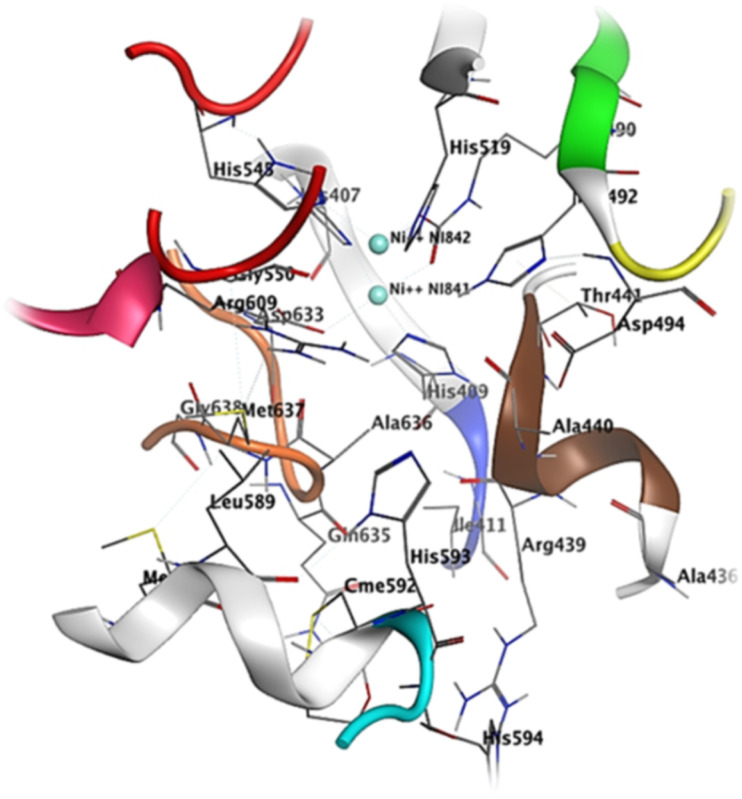
Active site residues including sheet S23 (residues 403–409, color coded silver) a small loop L30 (410–412, blue), helix H14 (435–441 color coded brown), loop L37 (444–454 color coded red), helix H20 (580–593 color coded green), loop L39 (594–598 color coded cyan), helix H21 (599–610 color coded pink) and a loop L41 (632–638 color coded orange).

We performed molecular docking study to evaluate the tendency of compounds identified from *C. mimosides* as potential inhibitor for urease enzyme. The protein (PDB ID: 3LA4) and ligands prepared as described in Materials and methods were docked into the active site of the urease enzyme. The result of this calculation is shown in Table 4, while the docking results are shown in Fig. 7, and S9.† The docking analysis results have provided valuable insights into the binding affinities and interactions between specific compounds and the 3LA4 protein. Among the compounds tested, 16, 6, 5, 1, and 7 demonstrated the highest binding affinities, with energy values of −7.43, −7.68, −8.31, −8.79, and −6.79 kcal mol^−1^, respectively. Conversely, compound 4 exhibited the weakest binding interaction, with a binding affinity of −7.84 kcal mol^−1^, indicating a lower likelihood of effective binding. The majority of protein–ligand complexes displayed the presence of hydrogen bonds, which play a pivotal role in stabilizing these interactions. However, some compounds, specifically 1, 8, 9, and 13, did not form hydrogen bonds with certain key amino acid residues, including Ala80, Asp295, Asp730, Glu34, Glu742, Gly714, Gly641, Ile148, Phe838, Ser421, and Tyr309. This absence of hydrogen bonds in these cases might influence the overall binding and stability of the complexes.

**Table 4:** Binding score and RMSD from protein urease

Compound name	Binding score (kcal mol^−1^)	RMSD (Å)
Luteolin-6-C-β-d-glucopyranoside (7)	−6.79	1.49
Luteolin-8-C-β-d-glucopyranoside (6)	−7.68	1.80
Apigenin-8-C-β-d-glucopyranoside (5)	−8.31	2.09
Nonadecanoic acid (14)	−8.47	1.88
Luteolin-5-methyl ether (4)	−7.84	1.53
Luteolin (2)	−8.61	0.99
β-Sitosterol 3-O-β-d-glucopyranoside (16)	−7.43	1.84
Butin (8)	−7.38	1.67
β-Sitosterol (15)	−6.97	1.37
Stigmat-4-en-3,6-dione (9)	−6.19	1.26
21-Methylene-24-ethylidene lophenol (1)	−8.79	1.19
Physine (13)	−5.84	1.45
Emodin (11)	−5.59	1.84
Chrysophanol (12)	−5.51	1.71
3′,4′,7-Trihydroxyflavone (3)	−8.49	1.32
Stigmast-4-en-3β,6α-diol (10)	−7.05	1.72
Reference (thiourea)	−3.13	2.15

The crystal structure of urease reveals crucial residues that form the binding pocket, facilitating the retention of inhibitors within the active site. These include a Ni-metal atom, sheet S23 (residues 403–409), a compact loop L30 (410–412, shown in blue), helix H14 (435–441 in a brown color), loop L37 (444–454 in red), helix H20 (580–593 in green), loop L39 (594–598 in cyan), helix H21 (599–610 in pink), and a loop L41 (632–638 in orange). Compound 1 establishes a binding interaction with the urease active site, displaying a binding energy of −8.79 kcal mol^−1^. Notably, compound 1 possesses alkyl and olefinic moieties that enable interactions with the active pocket, leading to several pi–alkyl interactions observed with key residues in the active site, particularly with His549, Ala436, and Ala440. Compound 2 effectively binds to the urease active site, exhibiting a binding energy of −8.61 kcal mol^−1^. This interaction involves both nickel atoms within the active site, leading to the formation of a protein–inhibitor complex. Notably, conventional hydrogen bonds are established with amino acids Asp633, Gly550, Ala440, and H14. Furthermore, a π–alkyl interaction occurs between compound 2 and Met637, while a π–π interaction is observed between His519 and the compound. The binding of compound 7 to the urease active site is robust, displaying a binding energy of −6.79 kcal mol^−1^. This interaction centers around the active Ni-metal atom, facilitated by the hydroxyl group of the sugar moiety. Conventional hydrogen bonds are prominent, involving amino acid residues Arg436, Ala440, Arg609, and Glu493. Additionally, π–cation interactions are established with Met637, His593, and Asp494. Interestingly, a majority of the studied compounds exhibited enhanced accuracy in the docking protocol compared to the reference compound, with root-mean-square deviations (RSMDs) ranging from 0.63 to 1.79 Å. This indicates that the majority of the studied compounds had relatively precise docking predictions, reinforcing the reliability of the docking protocol for evaluating their binding interactions with the 3LA4 protein.

### 2.15 Drug similarity analyses and ADME studies

Similarity analyses with reference compounds and ADME predictions for the natural compounds studied were performed *via* the SwissADME web server.^8^ The results are presented in Tables 1S and 2S† In these analyses, no secondary metabolites studied had more than twelve hydrogen bond acceptors. However, compounds 5, 6 and 7 have the highest number of hydrogen bond acceptors with values between ten and eleven. On the other hand, although compounds 5, 6 and 7 obey the Lipinski rule while possessing more than five hydrogen bond donors. The molecular masses of the natural compounds studied are between 254.24 and 576.85 g mol^−1^.^9,10^ The drug character of a given molecule was predicted using topological polar surface area (TPSA).^10^ From these results, it can be seen that, the polar surface area of the majority of the studied compounds are less than 111.13 Å^2^.

The water solubility of the studied secondary metabolites (ESOL, ALI and SILICOS-IT^16^) varies from poorly soluble to soluble. The high value of gastrointestinal absorption of compound 12 may therefore imply that it can cross the blood–brain barrier. We were also able to observe a variation in skin permeability −1.91 to −9.14 cm s^−1^.^17^ These results also show that all the molecules obeyed the rules of Lipinski (86.66%), Ghose (53.33%), Veber (73.33%), Egan (53.33%) and Muegge (46.66%), and Abbott's bioavailability scores^18^ were predicted at 0.55 for 80% of the molecules studied.

#### 2.15.1 % Viability of synthesized compounds against human HepG2 hepatic cancer cells

Human HepG2 hepatic cancer cells viability of natural product isolates summarized in Table 5, which shows that the compound 9 is highly potent against human HepG2 liver cancer cell lines; the compounds *i.e.*, 9 and 10 also show good response as their percent viability is 31.13 and 14.47 respectively at 200 μg. These compounds fit into the pocket at the specific site and effectively inhibit the growth of cancer cells. The compound shows the best response against human HepG2 liver cancer cell lines *i.e.*, % viability of compound 10 is 14.47% ± 0.69 and IC_50_ is 7.8 μM ± 0.21 (calculated using Graph Pad Prism software). Therefore, it can be deduced that the isolated compounds are highly potent molecules as compared to the previously reported ones. Moreover, these compounds are more potent inhibitors of HepG2 cell line than reported standard (thalidomide). Compound 10 is considered to be more potent than the reported analogue of phthalimide.

**Table 5:** % Viability and standard deviation of isolated compounds against HepG2 cancer

Sample	% Viability
Chrysophanol (12)	59.32 ± 2.20
Physine (13)	87.44 ± 2.55
β-Sitosterol 3-O-β-d-glucopyranoside (16)	96.40 ± 2.20
Luteolin (2)	82.46 ± 4.31
β-Sitosterol (15)	94.44 ± 4.42
Stigmat-4-en-3,6-dione (9)	31.13 ± 2.68
Stigmast-4-en-3β,6α-diol (10)	14.47 ± 0.68
Emodin (11)	83.89 ± 0.73
Apigenin-8-C-β-d-glucopyranoside (5)	94.67 ± 7.40
Lutéolin-8-C-β-d-glucopyranoside (6)	89.23 ± 2.50
Luteolin-5-methyl ether (4)	90.55 ± 2.15
Luteolin-6-C-β-d-glucopyranoside (7)	97.94 ± 3.53

## 3. Methodology

### 3.1. General experimental procedures

Acetone-d6, chloroform-d, metthanol-d4, and DMSO-d6 were used as the analytical solvent for ^1^H and ^13^C NMR spectra (Bruker AMX-400 MHz and a Bruker Avance III-600 MHz). Tetramethylsilane (TMS) was used as a reference to the solvent residue while coupling constants (*J*) and chemical shifts (*δ*) are expressed in Hz and parts per million units (ppm) respectively. Silica gel 60 (0.040–0.063 mm, Merck), 60 F254 (Merck), OSD, and Sephadex LH-20 gel were used in different chromatographic techniques (HPLC, column, and TLC). Sulfuric acid 10% and UV light (254 and 365 nm) were used to reveal different secondary metabolites.

### 3.2. Plant material

The aerial part of *C. mimosoïdes* was collected in Dschang (5°27′0′′N, 10°4′0′′E), West Region of Cameroon, in October 2018 and identified at the National Herbarium of Cameroon (NHC), Yaounde, where a voucher specimen (No. 8521/HCN) was deposited.

### 3.3. Extraction and isolation of the plant constituents

The aerial part of *C. mimosoïdes* was cut, dried and ground to give 6 kg of powder. 4 kg of this powder was extracted with ethanol (20 L) 3 × 24 h at room temperature followed by filtration. The filtrate obtained was concentrated under reduced pressure to give a crude extract (141 g). The extract was suspended in water and successively partitioned to ethyl acetate and butanol fractions. The ethyl acetate and *n*-butanol fractions were subjected to silica gel column chromatography using different solvent mixtures (*n*-hexane–ethyl acetate, ethyl acetate–methanol and ethyl acetate–methanol–water) in increasing polarity to give twelve subfractions (A-M). By repeating different chromatographic techniques (column and HPLC) of these twelve fractions, one new compound (1) and fifteen known secondary metabolites (2–16) were obtained (Fig. 1). It should be noted that all of these known compounds are reported herein for the first time from *Cassia mimosoïdes*.

### 3.4. Synthesis of semi-synthetic derivative (10) from stigmast-4-en-3,6-dione

In a 50 mL flask, 20 mg (4.69 × 10^–5^ mol) of stigmast-4-en-3,6-dione (10) was dissolved in 6 mL of methanol and refluxed under stirring until complete dissolution and then allowed to cool (25 °C). While under continuous stirring, 2 mg sodium borohydride (NaBH_4_) previously dissolved in 3 mL distilled water was added drop-wise and the evolution of the reaction was monitored by TLC until complete disappearance of starting material (20 min) as illustrated in Fig. 2. The reaction medium was then transferred to a beaker (250 mL) containing 50 mL of acidified ice-cold distilled water (3 mL of concentrated HCl). After 20 minutes the formation of a precipitate was observed. The medium was then filtered to yield 14.8 mg or 73.3%.

### 3.5. *In vitro* studies

#### 3.5.1. Antifungal bioassays

*Alternaria alternata*, *Aspergillus fumigatus*, *Aspergillus niger*, and *Penicillium citrii* strains were obtained from the Biotechnology lab at COMSATS University Islamabad. The antifungal activity of isolated compounds was assessed using poisoned medium technique. Two percent malt extract agar medium was generated by autoclaving at 121 °C for 30 minutes. Weighed volumes of the compounds were dissolved in 500 mL of sterilized distilled water and put to flasks containing 59.5 mL of malt extract agar medium while they were still molten to obtain final concentrations of 100, 300, 500, 700, 900, and 1000 ppm. The control received the same amount of distilled water. Each sterile Petri plate with a diameter of 90 mm was filled with 20 mL of each medium and allowed to solidify. 5 mm diameter mycelial discs were produced from the tips of 5–7 day-old cultures of the four test fungus species and put in the center of each Petri dish using a sterile cork borer. Every treatment was duplicated three times. Plates were cultured in an incubator at 25 ± 2 °C for seven days. To calculate the rate of fungal growth, the three diameters of each colony were measured at right angles and averaged. Table 2 MIC values (μM) of the isolated compounds against *Alternaria alternata*, *Aspergillus fumigatus*, *Aspergillus niger*, and *Penicillium citrii* strains.^19^

#### 3.5.2. Antibacterial activity

The agar well diffusion method evaluated every compound separately. Each sterile Petri plate was aseptically filled with 20 mL of the agar medium, which was then autoclaved at 120 °C to destroy any germs. The media then solidified at room temperature. Various bacterial strains were put to agar plates using a sterile cotton swab. A 6 mm-diameter well was drilled using a sterilized cork borer. Each isolated compound was dissolved in 1 mL of a 20% DMSO solution. In a well with a diameter of 6 mm, 20 mL of the standard drug and isolated compounds were added. At 37 °C, the test plates were incubated for 24 hours. The test plates were incubated at 37 °C for 24 hours. The standard disc containing 50 mg of ciprofloxacin served as a positive control for antibacterial activity, and DMSO served as a negative control. The zone of inhibition was graphed and quantified in millimeters.^20^

#### 3.5.3. Urease activity test

In 96-well plates were incubated at 37 °C for 15 min, reaction mixtures consisting of 25 μL of test compounds (each 0.5 mM), 25 μL of enzyme solution (Jack bean urease) and 55 μL of buffers containing 20.79 mM urea. The indophenol method described by Weatherburn^21^ was used to determine urease inhibitor activity by measuring ammonia production. Briefly, to each well were added 70 μL of alkaline reagent (0.5% w/v NaOH and 0.1% active chloride, NaOCl) and 45 μL of phenolic reagent (1% w/v phenol and 0.005% w/v sodium nitroprusside). The microplate reader (Molecular Devices, USA) was used to measure the increase in absorbance at 650 nm after 50 min. A final volume of 245 μL was required to perform all reactions in a triplicate. The results (absorbance change per minute) were processed using SoftMax Pro software (Molecular Devices, USA). All assays were performed at pH 6.8. Inhibition percentages were calculated from the formula% Inhibition = 100 − (Abs_(sample)_/Abs_(control)_) × 100where “*A*” is the absorbance of the “test well” as well as the “control”. Thiourea was used as a standard.

#### 3.5.4. HEPG2 cancer cell lines – cell culture and treatment

The human liver cells were cultivated in Dulbecco's modified Eagle medium (DMEM) in which 100 U mL^−1^ penicillin, 10% fetal bovine and 100 g mL^−1^ streptomycin were added and kept at 37 °C in a 50% carbon dioxide induced humidified environment. The human liver cells prepared as per above procedure were then treated with synthesized compounds diluted to 0.05% DMSO concentration.

#### 3.5.5. Determination of cell viability

Using MTT assay, the % viability of cancer cells 36 was evaluated. According to the standard procedure, HepG2 cells were treated with various dosages of synthesized compounds for 48 hours each. The cells were then incubated for 4 hours at 37 °C with 10μ MTT reagent (5 mg mL^−1^). Next, in 150 μL DMSO was added to dissolve formazan crystals. The *λ*_max_ was recorded at 409 nm in a microplate reader (thermos scientific).

#### 3.5.6. Molecular docking study

ChemDraw Professional 15.0 was used to draw the structures of the isolated secondary metabolites, and MOE (Molecular Operating Environment) software (version 2019.0102) was used for molecular docking. Molecular docking using MOE (Molecular Operating Environment) is a computational technique used to predict the binding interactions between a ligand (small molecule) and a target receptor (protein). Urease crystal structure with the PDB ID 4GOA, extracted from protein databank, has been used to study protein–ligand interactions.^22^ The 3D structures of both the ligands and the protein were prepared following addition of hydrogen atoms, assigning partial charges, and optimizing the structures. A grid is generated around the binding site of the protein. This grid defines the search space for possible ligand binding orientations. MOE employs a scoring function to estimate the binding energy between the ligand and the protein. For each ligand pose, the scoring function calculated the binding energy. The poses were ranked based on their energy scores, with lower scores indicating more favorable binding affinity. After the docking simulation was completed, the generated 2D and 3D interaction poses were analyzed to understand the potential binding mode and interactions involving between the ligand and the protein.^8^ Various pharmacokinetic parameters such as physicochemical properties, lipophilicity and water solubility were predicted in this study.

## 4. Conclusions

All the compounds showed appreciable antimicrobial activity against selected strain of bacterial and fungal origin. Compound 5 and 6 showed moderate to good antifungal activity against all selected strains. The genus *Cassia* is a significant source of secondary metabolites that are physiologically active and come from several chemical classes. The current research discusses the spectroscopic elucidation of the structure and enzymatic activity *in silico* and *in vitro* of fifteen known compounds as well as a new unidentified avenasterol derivative called 21-methylene-24-ethylidene lophenol. The compound isolated from C. mimidosa holds significant potential as a drug candidate for treating illnesses associated with *H. pylori* infection. This potential arises from its demonstrated effectiveness in targeting the urease enzyme and its promising activity against the HEP-G2 cell line. The urease enzyme is a key factor in the survival of *H. pylori* within the stomach's acidic environment. Inhibiting urease activity can potentially impair the bacterium's ability to colonize and cause damage. The compound from *C. mimidosa* has shown strong inhibitory activity against the urease enzyme, suggesting its potential to disrupt *H. pylori*'s survival mechanism. Compound 1 showed strong antibacterial activity against *H. pylori* and *S. aur* with MIC value of 0.28 and 0.12 respectively. The inhibitory activity of all the substances tested against urease was higher (1.224 ± 0.43 IC_50_ > 6.678 ± 0.11 M) than that of thiourea (IC_50_ = 18.61 ± 0.11 M). The compound's 10 and 9 positive effects on the HEP-G2 cell line further underscore their potential as a drug candidate. The HEP-G2 cell line is often used as a model to study the effects of potential drugs on cancer cells. The compound's activity against these cells indicates that it might have a broader impact on cellular processes, potentially offering therapeutic benefits beyond its anti-*H. pylori* effects. In conclusion, the compound isolated from *C. mimidosa* shows great promise as a multi-faceted drug candidate for treating illnesses associated with *H. pylori* infection. Its inhibition of urease enzyme activity suggests a targeted approach against *H. pylori*, while its positive effects on the HEP-G2 cell line hint at broader applications, possibly including anti-cancer properties. Further research and clinical studies are warranted to fully explore the compound's potential as a novel therapeutic agent.

## Conflicts of interest

The authors declare no conflict of interest.

## Supplementary Material

RA-014-D3RA08913D-s001
