# Supplementary material for: Exploring Cassia mimosoïdes as a promising natural source of steroids with potent anti-cancer, urease inhibition, and antimicrobial properties
Source: RSC Adv. 2024 Mar 18;14(13):9159–68. doi: 10.1039/d3ra08913d (PMC10945739; doi:10.1039/d3ra08913d)
Supplement: RA-014-D3RA08913D-s001 [file RA-014-D3RA08913D-s001.pdf]

Electronic Supplementary Material (ESI) for RSC Advances.
This journal is © The Royal Society of Chemistry 2024

Exploring Cassia mimosoïdes as a Promising Natural Source of Steroids with Potent Anti-Cancer, Urease Inhibition, and Antimicrobial Properties

Robert Viani Kepdieu Tchebou[1,2], Umar Farooq[2,3*], Azhar Rasool[4], Tanveer A. Wani[5], Seema Zargar[6], Rizwana Sarwar[2], Aneela Khushal[2], Léon Azefack Tapondjou [1], Syed Majid Bukhari[2], Seema Zargar[6], Rémy Bertrand Teponno[1], Hong-Guang Xu[3], Sara Khan[2,*]

Contents

Fig. S1. SAS-RS-APCI spectrum of compound **1**

Fig. S2. ^{1}H NMR spectrum of compound **1**

Fig. S3. ^{13}C NMR spectrum of compound **1**

Fig. S4. DEPT90 spectrum of compound **1**

Fig. S5. ^{1}H DEPT 135 spectrum of compound **1**

Fig. S6. Superposition of DEPT 90, DEPT 135 and ^{13}C NMR spectrum of compound **1**

Fig. S7. ^{1}H-^{1}H COSY spectrum of compound **1**

Fig. S8. HSQC spectrum of compound **1**

Fig. S9. HMBC spectrum of compound **1**

Fig. S10. IC_{50} of compound **2, 4, 5, 7, 10, 11, 12, 14 and 15**

Fig. S11. 2D and 3D view of binding interaction of β-glucosidase and compound **1**

Fig. S12. 3D view interaction hydrophobicity of compounds **1**

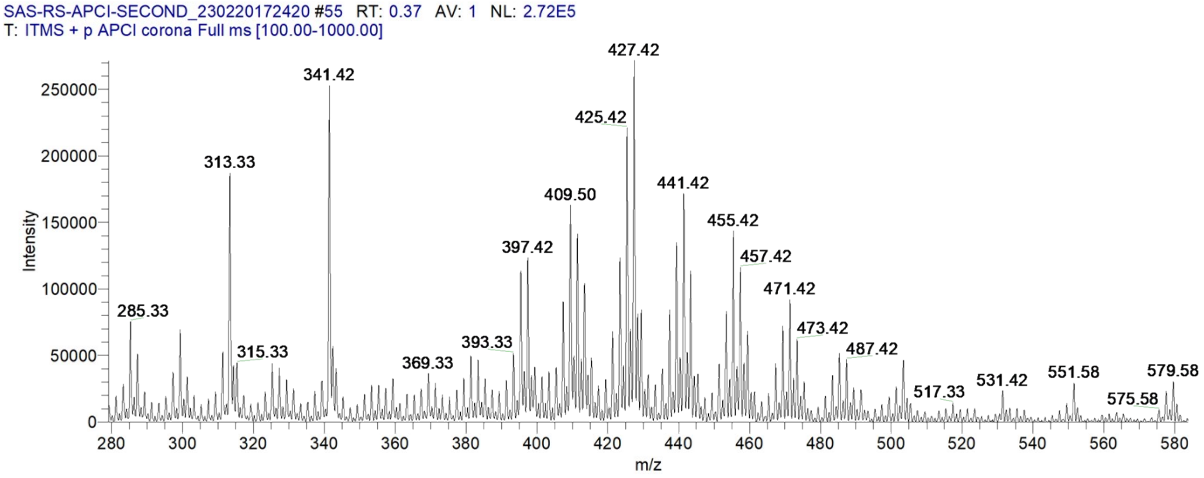

Fig. S1. SAS-RS-APCI spectrum of compound **1**

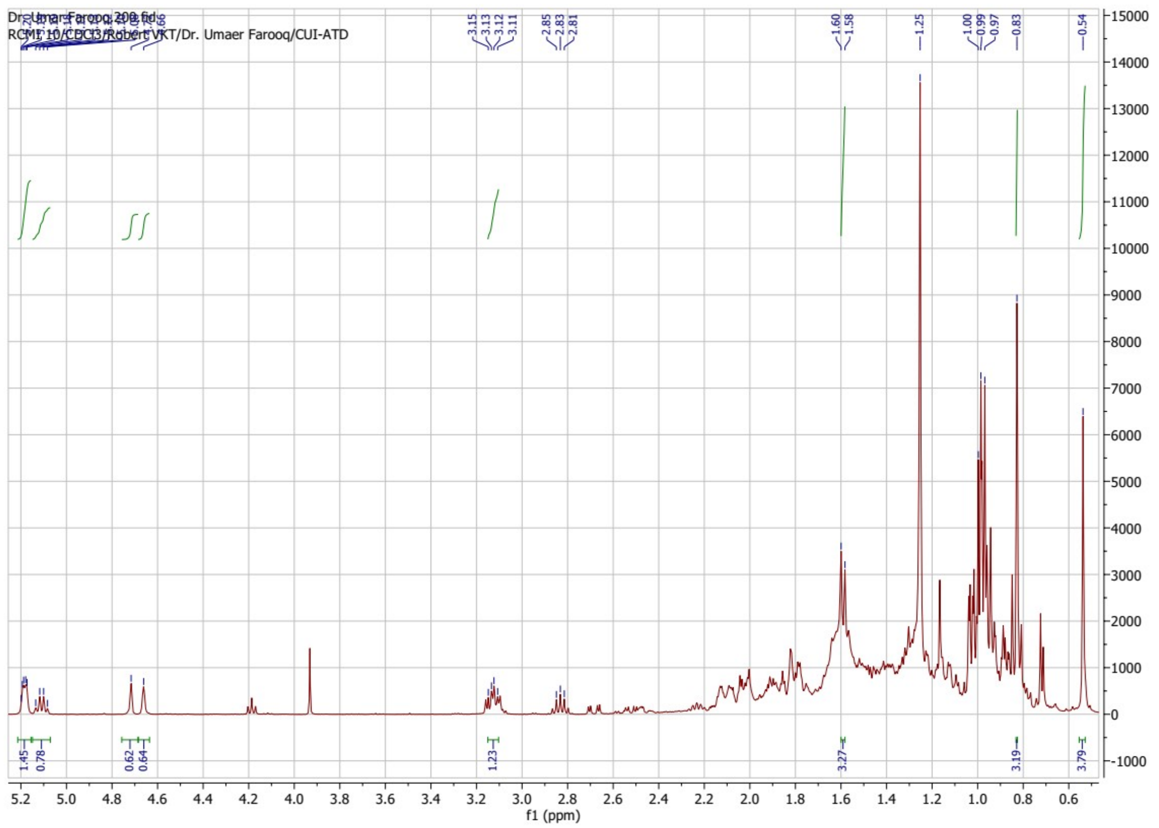

Fig. S2. ^{1}H NMR spectrum of compound **1**

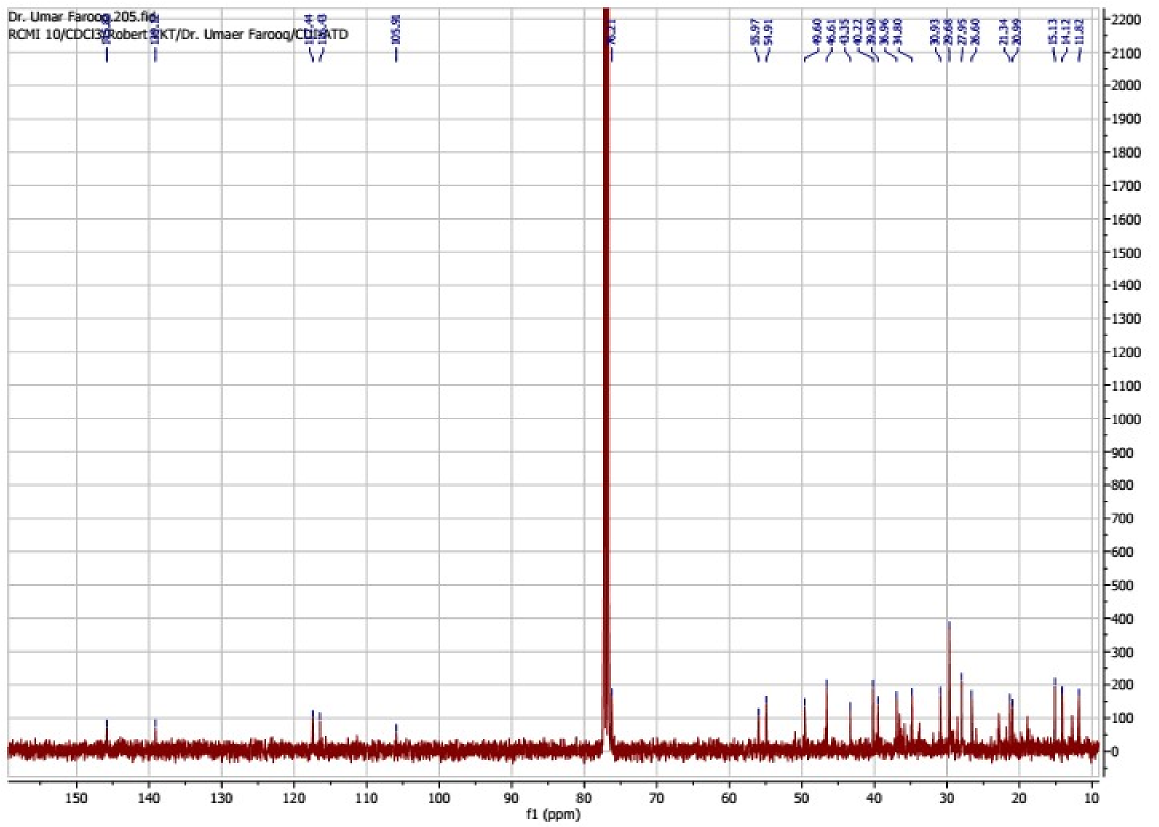

Fig. S3. ^{13}C NMR spectrum of compound **1**

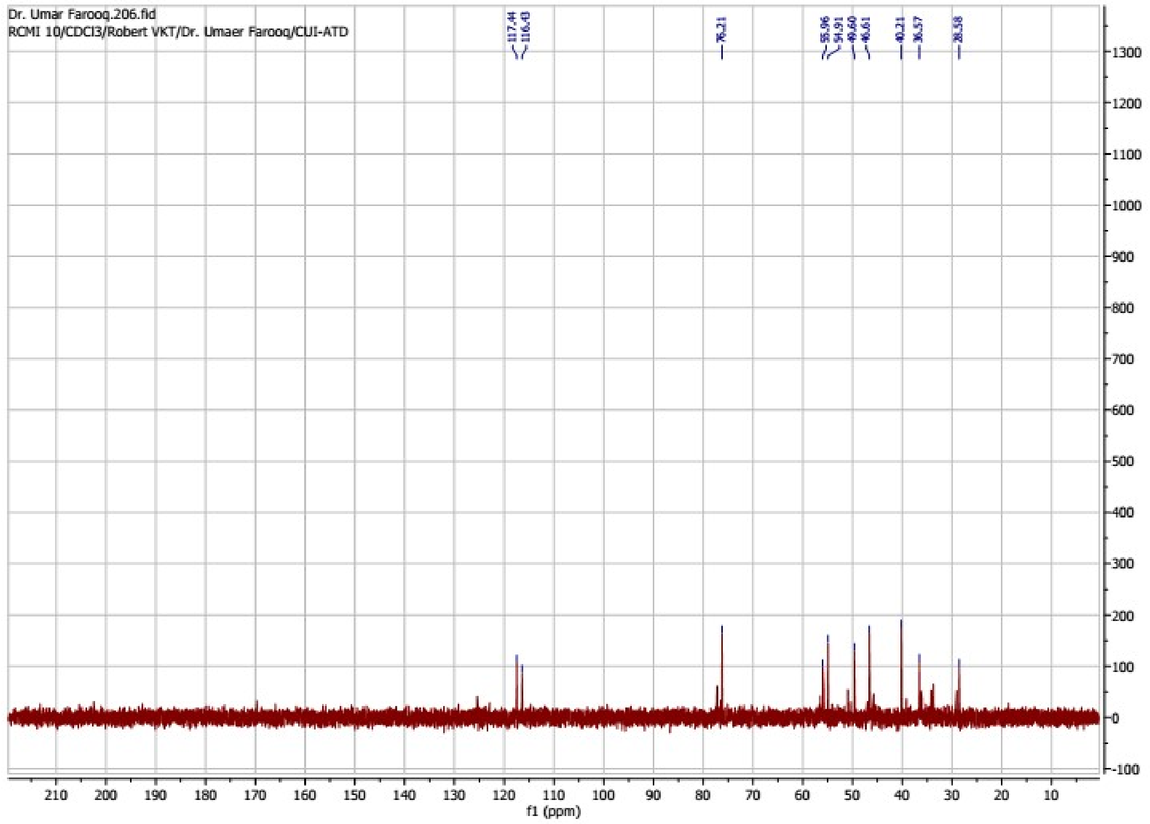

Fig. S4. DEPT90 spectrum of compound **1**

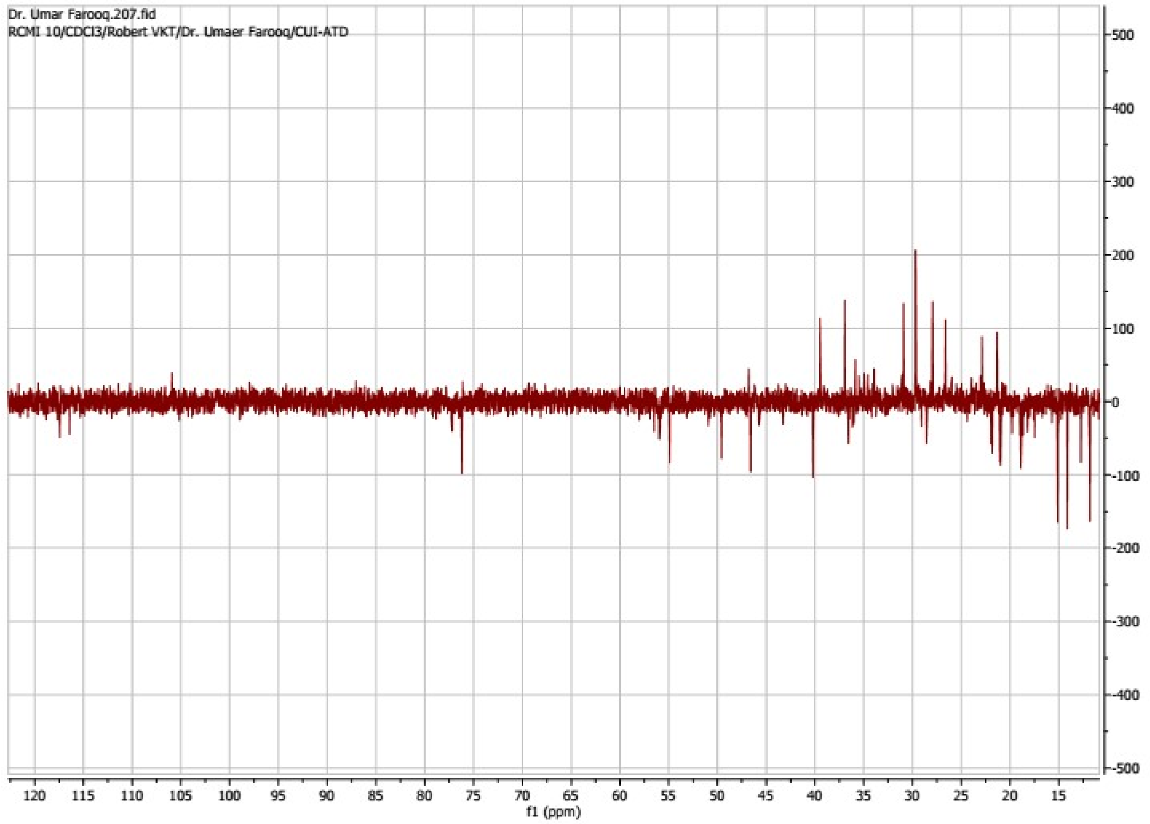

Fig. S5. ^{1}H DEPT 135 spectrum of compound **1**

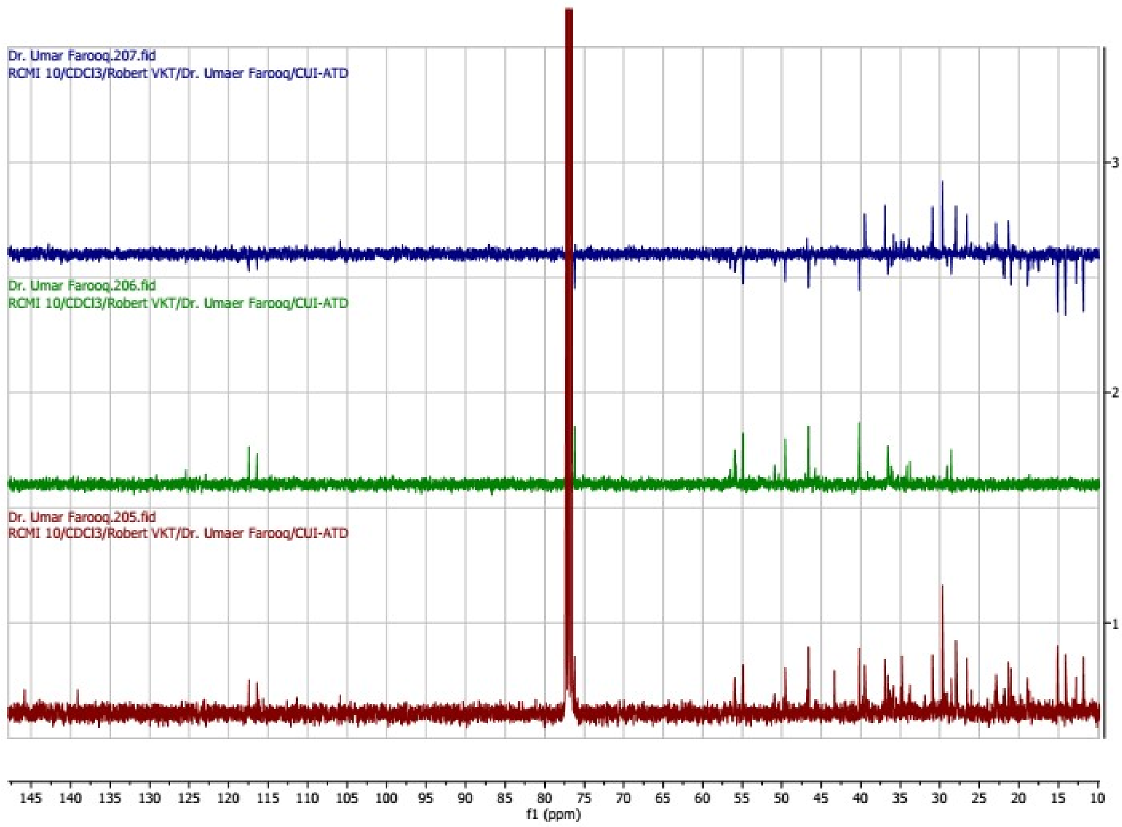

Fig. S6. Superposition of DEPT 90, DEPT 135 and ^{13}C NMR spectrum of compound **1**

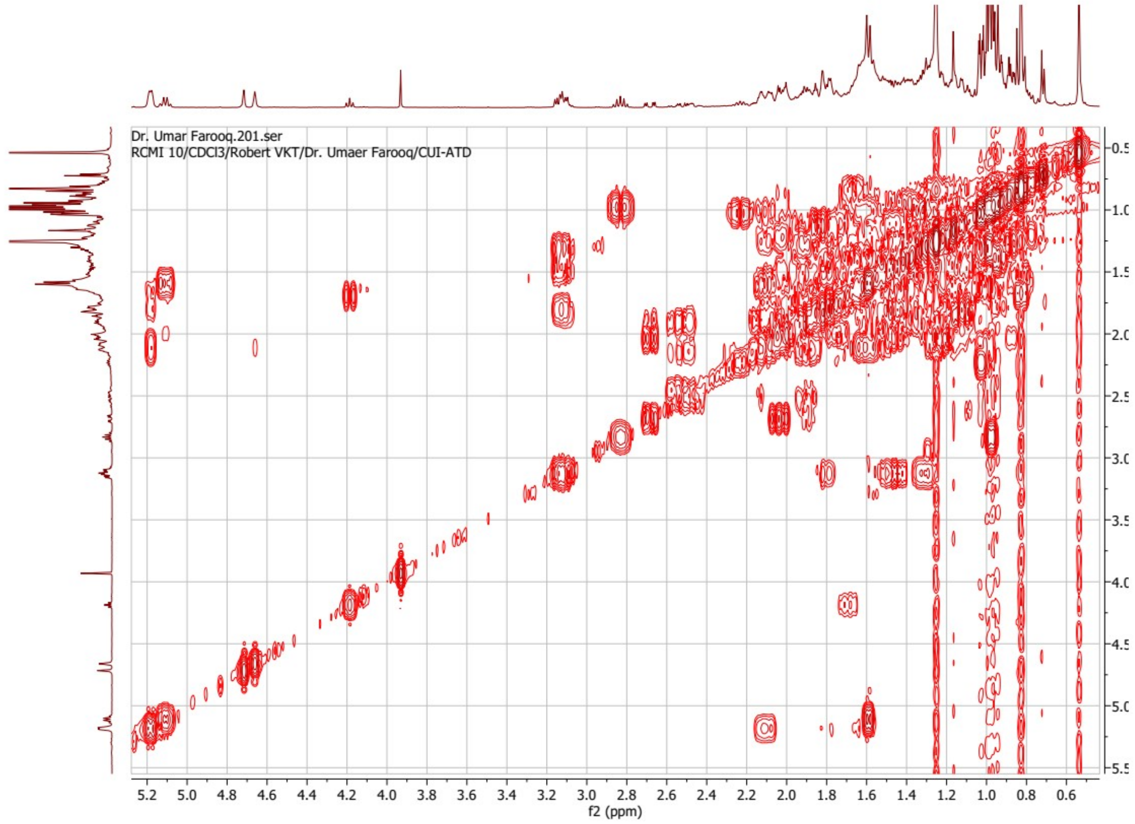

Fig. S7. ^{1}H-^{1}H COSY spectrum of compound **1**

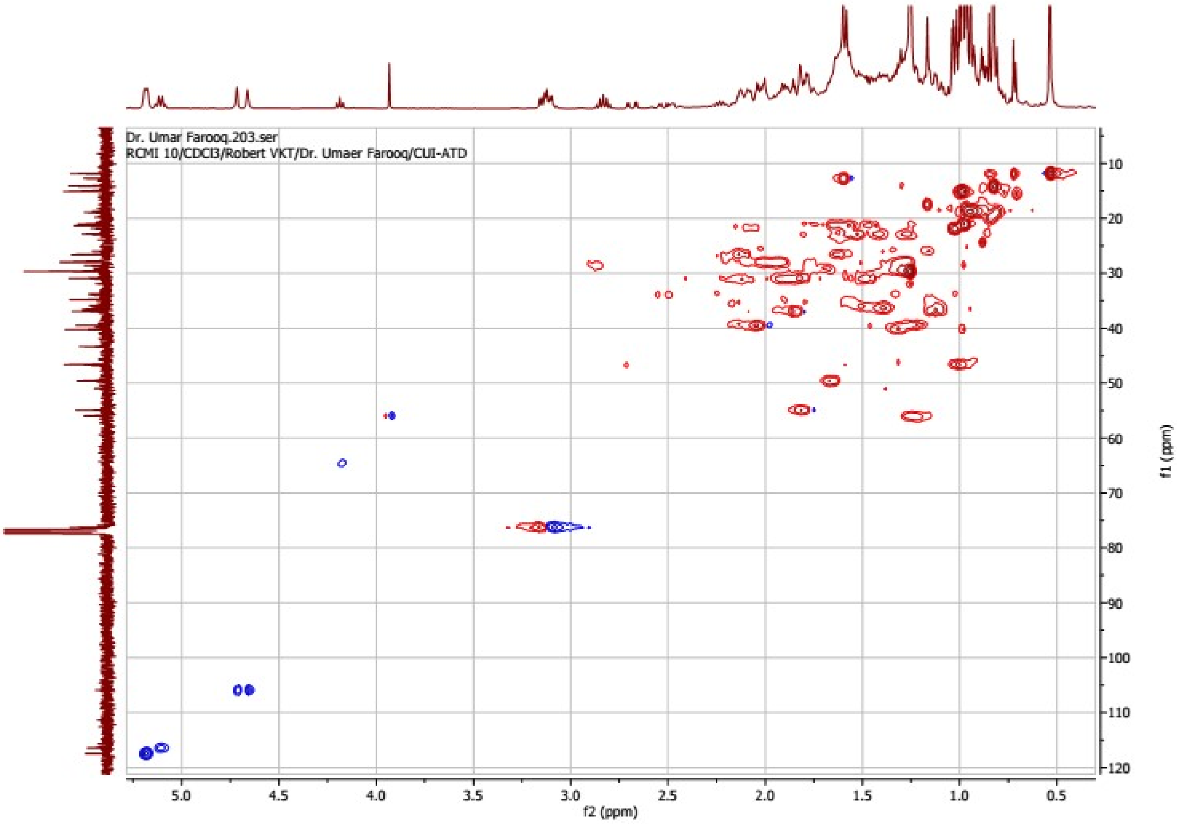

Fig. S8. HSQC spectrum of compound **1**

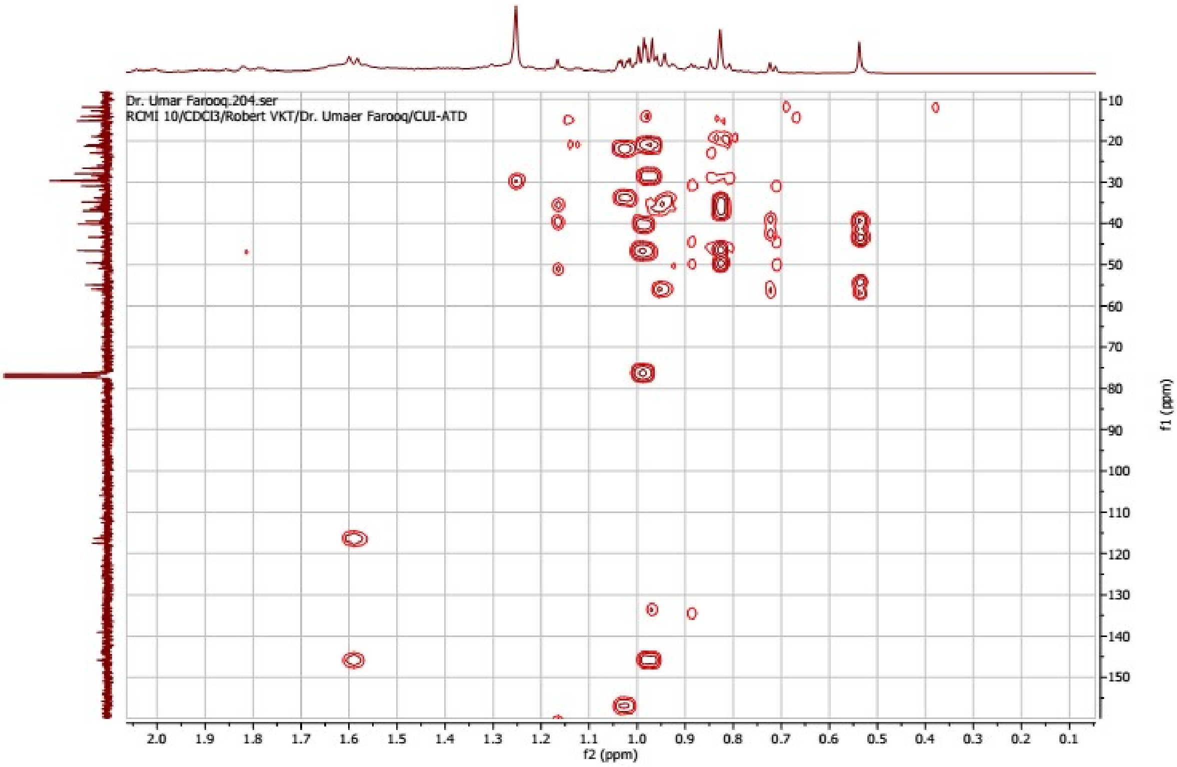

Fig. S9. HMBC spectrum of compound **1**

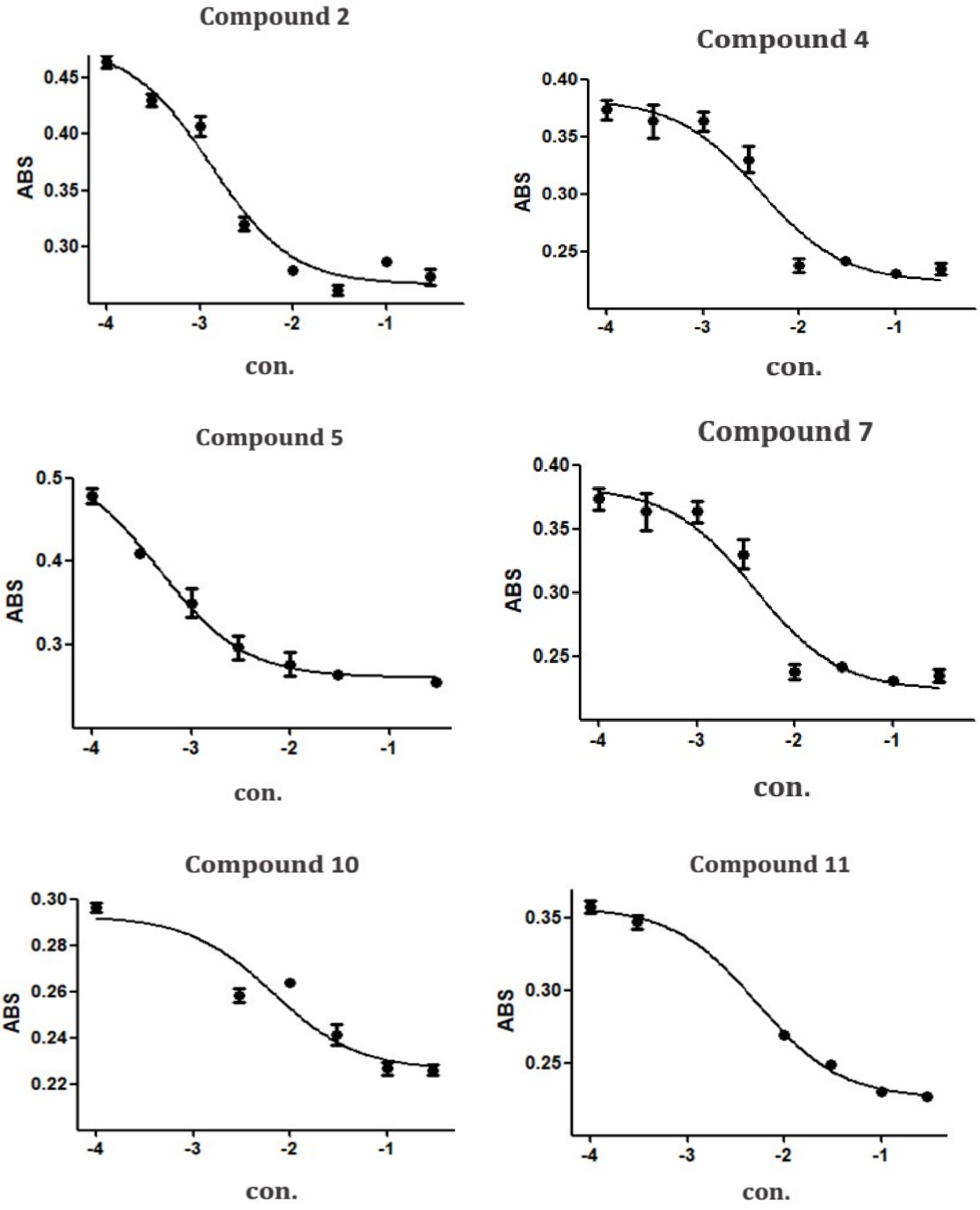

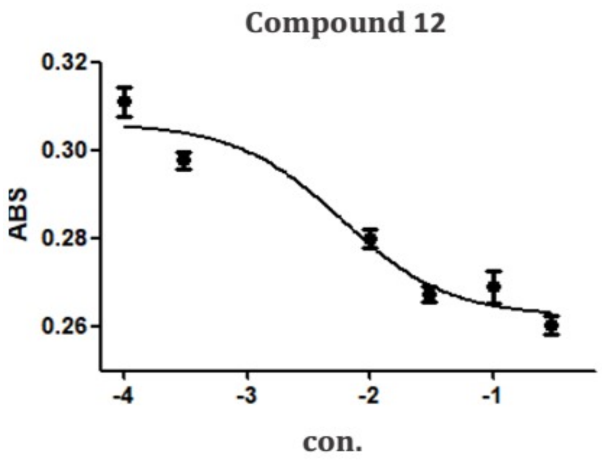

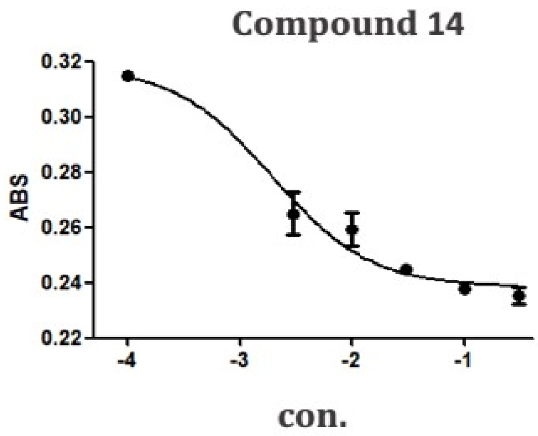

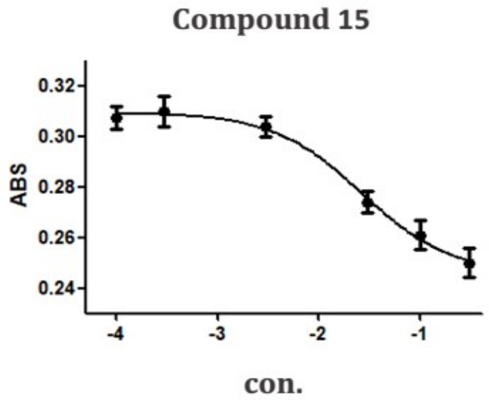

Table S1: Binding score and RMSD from urease

Compound Name	Binding score (kcal/mol)	RMSD (Å)
**Luteolin-6-*C*-*β*-D-glucopyranoside(7)**	-8.79	1.49
**Luteolin-8-C-β-D-glucopyranoside (6)**	-8.68	1.80
**Apigenin-8-*C*-*β*-D-glucopyranoside (5)**	-8.31	2.09
**Nonadecanoic acid (14)**	-8.47	1.88
**Luteolin-5-methyl ether (4)**	-7.84	1.53
**Luteolin (2)**	-7.61	0.99
***β*-sitosterol 3-*O*-*β*-D-glucopyranoside (16)**	-7.43	1.84
**Butin (8)**	-7.38	1.67
β*-sitosterol (15*)	-6.97	1.37
**Stigmat-4-en-3,6-dione (9)**	-6.19	1.26
**21-methylene-24-ethylidene lophenol (1)**	-6.05	2.19
**Physine (13)**	-5.84	1.45
**Emodin (11)**	-5.59	1.84
**Chrysophanol (12)**	-5.51	1,71
**3',4',7-trihydroxyflavone (3)**	-5.49	2.32
**Stigmast-4-en-3*β*,6*α*-diol (10)**	-7.05	1.72
**Standard (thiourea)**	-3.13	2.15

Table S2: Antibacterial activity of isolated compounds against bacterial strains**.** *H.pylori*, *B.subtilis* and *S.aureus*

Compounds	*H.Pylori*	*S. aureus*	*B. subtilis*
**7**	1.23	0.78	0.12
**2**	0.21	1.34	0.95
**14**	0.23	2.67	0
**15**	0.95	0.234	0
**4**	0.52	0.42	1.76
**5**	3.24	0.678	2.67
**11**	1.2	0.82	1.78
**12**	3.45	1.78	1.56
**10**	2.24	2.83	0.67
**1**	0.28	0.12	0
**Synthesized compound**	0.24	0.89	0.16
**6**	2.64	3.56	0.19
**9**	0	2.45	0.11
**13**	3.43	1.56	0.17
**16**	2.3	1	1.1
**Ciprofloxacin**	0.67	0.187	0.75

Table S3 Results of ADME analyses of the investigated compounds.

**Compounds**	**1**	**2**	**3**	**4**	**5**	**6**	**7**	**8**	**9**	**10**	**11**	**12**	**13**	**14**	**15**	**16**	**THI**	**ACE**	**ACA**
Physicochemical Properties																			
Number of H-Bond Acceptors	1	6	5	6	10	11	11	5	2	2	5	4	5	2	1	6	0	6	19
Number of H-Bond Donors	1	4	3	3	7	8	8	3	0	2	3	2	2	1	1	4	2	2	14
Molecular Weight (g/mol)	424.70	286.24	270.24	300;26	432.38	448.38	448.38	272.25	426.67	430.71	270.24	254.24	284.26	298.50	414.71	576.85	76.12	222.25	645.60
Number of heavy atoms	31	21	20	22	31	32	32	20	31	31	20	29	21	21	30	41	4	13	44
Number of aromatic heavy atoms	0	16	16	16	16	16	16	12	0	0	12	12	12	0	0	0	0	5	0
Fraction C sp3	0.80	0.00	0.00	0.06	0.29	0.29	0.29	0.13	0.86	0.93	0.07	0.07	0.12	0.95	0.93	0.94	0.00	0.25	0.92
Number of rotatable bonds	5	1	1	2	3	3	3	1	6	6	0	0	1	17	6	9	0	3	9
Molar refractivity	137.09	76.01	73.99	80.48	106.61	108.63	108.63	71.57	132.47	134.39	70.78	68.76	75.25	95.22	133.23	165.61	20.13	45.22	136.69
TPSA (Å^2)	20.23	111.13	90.90	100.13	181.05	201.28	201.28	86.99	34.14	40.46	94.83	74.60	83.83	37.30	20.23	99.38	84.13	151.66	321.17
Lipophilicity																			
Ilogp	5.08	1.86	1.70	1.79	1.38	1.27	2.12	1.30	4.49	4.72	1.80	2.22	2.45	4.43	5.12	4.87	0.47	-0.21	-0.35
XLOGP3	8.86	2.53	2.91	2.31	0.21	-0.15	-0.15	1.94	8.37	8.09	2.72	3.53	3.04	8.75	9.34	7.74	-0.83	-0.26	-8.53
WLOGP	8.11	2.28	2.58	2.59	-0.32	-0.53	-0.53	2.19	7.41	7.00	1.89	2.18	2.19	6.72	8.02	5.85	-0.81	0.03	-8.56
MLOGP	6.72	-0.03	0.52	0.22	-2.02	-2.51	-2.51	0.71	5.60	5.80	0.36	0.94	0.64	4.91	6.73	3.96	-1.53	-2.34	-6.94
SILICOS-IT	7.01	2.03	2.52	2.55	0.33	-0.14	-0.14	2.05	7.33	6.15	2.55	3.04	3.07	6.57	7.04	5.02	0.54	-0.33	-7.69
Consensus logPo/w	7.16	1.73	2.04	1.89	-0.07	-0.41	-0.24	1.64	6.64	6.36	1.87	2.38	2.27	6.28	7.25	5.49	-0.43	-0.62	-6.41
Water Solubility																			
ESOL	PS	S	S	S	S	S	S	S	PS	PS	S	MS	S	PS	PS	PS	HS	VS	HS
ALI	PS	MS	MS	MS	S	S	S	S	PS	PS	MS	MS	MS	PS	PS	PS	VS	S	HS
SILICOS-IT	MS	S	MS	MS	S	S	S	S	PS	MS	S	MS	MS	PS	PS	MS	S	S	S
Pharmacokinetics																			
GI Absorption	Low	High	High	High	Low	Low	Low	High	Low	Low	High	High	High	High	Low	Low	High	Low	Low
BBB Permeation	No	No	No	No	No	No	No	No	No	No	No	Yes	No	No	No	No	No	No	No
Skin permeation, logKp (cm/s)	-2.60	-6.25	-5.88	-6.49	-8.79	-9.14	-9.14	-6.58	-2.96	-3.18	-6.02	-5.34	-5.88	-1.91	-2.20	-4.32	-7.35	-7.84	-16.29

TPSA: Topological Polar Surface Area, GI: Gastrointestinal, BBB: Blood Brain Barrier, IS: Insoluble, PS: Poorly soluble, MS: Moderately soluble, S: Soluble, HS: Highly soluble, VS: Very soluble, THI: Thiourea, ACE: Acetazolamide, ACA: Acarbose

Table 2S Results of drug-likeness and medicinal chemistry analyses.

**Compounds**	**1**	**2**	**3**	**4**	**5**	**6**	**7**	**8**	**9**	**10**	**11**	**12**	**13**	**14**	**15**	**16**	**THI**	**ACE**	**ACA**
Drug-likeness																			
Lipinksi	Yes	Yes	Yes	Yes	Yes	No	No	Yes	Yes	Yes	Yes	Yes	Yes	Yes	Yes	Yes	Yes	Yes	No
Ghose	No	Yes	Yes	Yes	Yes	No	No	Yes	No	No	Yes	Yes	Yes	No	No	No	No	No	No
Veber	Yes	Yes	Yes	Yes	No	No	No	Yes	Yes	Yes	Yes	Yes	Yes	No	Yes	Yes	Yes	No	No
Egan	No	Yes	Yes	Yes	No	No	No	Yes	No	No	Yes	Yes	Yes	No	Yes	No	Yes	No	No
Muegge	No	Yes	Yes	Yes	No	No	No	Yes	No	No	Yes	Yes	Yes	No	No	No	No	No	No
ABS	0.55	0.55	0.55	0.55	0.55	0.17	0.17	0.55	0.55	0.55	0.55	0.55	0.55	0.85	0.55	0.55	0.55	0.55	0.17
Medicinal Chemistry																			
PAINS (alerts)	0	1	1	1	0	1	1	1	0	0	1	1	1	0	0	0	0	0	0
Brenk (alerts)	1	1	1	1	0	1	1	1	0	1	0	0	0	0	1	1	1	1	1
Leadlikeness	No (2)	Yes	Yes	Yes	No (1)	No (1)	No (1)	Yes	No (2)	No (2)	Yes	No (1)	Yes	No (2)	No (2)	No (3)	No (1)	No (1)	No (2)
SAS	6.21	3.02	2.94	3.14	5.12	5.17	5.04	3.05	6.04	6.45	2.57	2.47	2.69	2.65	6.30	8.02	1.28	3.00	7.34

ABS: Abbott Bioavailability Score, PAINS: Pan Assay Interference Structures, SAS: Synthetic Accessibility Score.
